# Can ancestry and morphology be used as surrogates for species niche relationships?

**DOI:** 10.1002/ece3.6390

**Published:** 2020-06-03

**Authors:** Friedrich W. Keppeler, Kirk O. Winemiller

**Affiliations:** ^1^ Department of Ecology and Conservation Biology Texas A&M University College Station TX USA

## Abstract

Species interactions are difficult to quantify, and, consequently, many studies have used species traits and phylogeny as proxies under an assumption of niche conservatism (i.e., closely related and morphologically similar species should have similar niches). However, few studies have investigated whether niches actually are conserved within and across diverse communities**.** Here, we tested the degree to which phylogenetic relatedness and morphological similarity predict diets and stable isotopic ratios (*δ* ^15^N and *δ* ^13^C), two common descriptors of the trophic niche, in fish assemblages of two small streams in the Neotropics. We also tested the strength of the association between isotopic ratios and diet composition and found significant correlations implying that isotopic signals reveal trophic structure despite error associated with estimates of trophic enrichment and variation associated with tissue preservation, metabolism, and other factors affecting isotopic ratios. Morphological traits yielded a significant phylogenetic signal, and both morphological traits and phylogeny were correlated with diet composition, with morphological traits being a stronger predictor. We infer that functionally relevant morphological traits of fish can be used to infer trophic niches for certain kinds of questions and analyses when trophic data are lacking. However, we highlight that using phylogenetic and morphological data in combination with dietary and/or isotopic data can improve resolution of assemblage trophic structure and niche diversification.

## Graphical abstract

We tested the degree to which phylogenetic relatedness and morphological similarity predict diets and stable isotopic ratios. Our findings reveal that both phylogeny and morphological traits were associated with fish diets and isotopic ratios; however, morphological traits were stronger predictors of dietary and isotopic ratios than phylogenetic relationships. We infer that functionally relevant morphological traits of fish can be used to infer trophic niches for certain kinds of questions and analyses when trophic data are lacking.

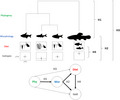

## 1 INTRODUCTION

Species interactions are an important mechanism structuring ecological communities (HilleRisLambers, Adler, Harpole, Levine, & Mayfield, 2012) with the potential to influence ecosystems processes and services (Traill et al., 2010). A fundamental challenge in ecology is to quantify these interactions and understand their implications for community assembly (Xu et al., 2018) and ecosystem dynamics (Jordano, 2016). In a broader context, a better understanding about species functional traits may improve understanding of evolutionary processes, such as adaptive radiation and convergence (Bower & Winemiller, 2019; Takahashi & Koblmüller, 2011). When analyses integrate functional, phylogenetic and species interaction data, diversity patterns can be elucidated and community assembly mechanisms can be inferred at multiple scales (Nanthavong et al., 2015; Peralta, 2016).

Direct and indirect metrics have been used to access species interaction strength (Berlow, Navarrete, Briggs, Power, & Menge, 1999; Wootton & Emmerson, 2005). From a food web perspective, stomach contents analysis (hereafter, referred to as dietary analysis) has been used to estimate predator–prey interactions (e.g., Rosi‐Marshall & Wallace, 2002) and infer the potential strength of interspecific competition (e.g., Jung, Stotyn, & Czetwertynski, 2015). Despite providing fairly direct documentation of consumer‐resource interactions, dietary analysis has some well‐known limitations, including (a) sample size dependency (i.e., a sample merely represents a snapshot in time and space, and may not reflect long‐term patterns of consumption); (b) difficulty to identify fragmented or partially digested food items; and (c) short retention time of ingested items (Araújo, Bolnick, Machado, Giaretta, & dos Reis, 2007; Votier et al., 2003). In recent decades, several studies have analyzed stable isotope ratios, especially of nitrogen (N) and carbon (C), as an alternative method for making inferences about trophic ecology (Fry, 2006). The ratio of ^15^N to ^14^N (*δ* ^15^N) is positively correlated with trophic level given its natural enrichment of 2–3‰ during assimilation of ingested material into consumer tissue (Peterson & Fry, 1987; Post, 2002). The ratio of ^13^C to ^12^C (*δ* ^13^C) varies among primary producers at the base of food chains and largely reflects differences in photosynthetic pathways (C3, C4, CAM) as well as inorganic sources of carbon assimilated by plants (Peterson & Fry, 1987). Consequently, variation in *δ* ^13^C and *δ* ^15^N of animals has been proposed as an indicator of trophic niche differences (Layman et al., 2012). A potential advantage of stables isotopes over diet analysis is its capability of integrating assimilation of consumed items over time (Layman et al., 2012), allowing the assessment of important ecological properties, such as individual specialization (Araújo et al., 2007; Harrison et al., 2017). Stable isotope ratios provide an indirect estimate of the trophic niche; however, isotopic ratios are influenced by other factors (Bastos, Corrêa, Winemiller, & Garcia, 2017; Davis, Blanchette, Pusey, Jardine, & Pearson, 2012; Villamarín et al., 2018; Zanden et al., 1997). For example, tissue isotopic turnover and trophic enrichment (Δ^13^C and Δ^15^N) can vary according to consumer body size, age, metabolism, and environmental conditions, which increases the uncertainty of estimates and inferences about trophic ecology based on stable isotopic analysis (Caut, Angulo, & Courchamp, 2009). In some cases, isotopic ratios may be more strongly associated with physiology linked to variation in morphological traits, such as body size, than with feeding history per se (Villamarín et al., 2018).

Morphologically similar species are generally expected to have similar niches (McGill, Enquist, Weiher, & Westoby, 2006; Rocha et al., 2011), resulting in relatively high dietary overlap (Gatz, 1979) and similar isotopic ratios provided that environmental conditions are similar (Hopkins & Kurle, 2016; Layman, Arrington, Montaña, & Post, 2007). Morphological traits often have a strong phylogenetic signal (Losos, 2008), and, therefore, one might expect a certain level of correlation between phylogenetic distance, diet, and isotopic ratios (Fraser, Haupt, & Barr, 2018; Lind, Vincent, Weiblen, Cavender‐Bares, & Borer, 2015). However, if there is rapid adaptive divergence or strong evolutionary convergence, some species may be more or less similar ecologically than would be expected based on phylogenetic relationships (Cachera & Le Loc'h, 2017; Kamilar & Cooper, 2013). Indicators of recent ecological performance, such as diet composition, would be expected to reveal weaker phylogenetic signals than morphological traits that have higher heritability, therefore less strongly influenced by environmental variation, and also are less subject to measurement error (Blomberg, Garland, & Ives, 2003; Freckleton, Harvey, & Pagel, 2002).

Trait‐diet relationships may be weak because species that appear to be specialists based on their morphology sometimes perform as ecological generalists under certain conditions (Liem, 1978); this incongruity has been called Liem's paradox (Greenwood, 1989; Mayr, 1984). A possible explanation for this paradox is that a given phenotype can perform multiple ecological functions, and different phenotypes sometimes can perform the same ecological function (Wainwright et al., 2005; Zelditch et al., 2017). For example, species that are trophic specialists during times of resource scarcity may switch to feed on profitable food resources when these are temporarily abundant (Robinson & Wilson, 1998). Similarly, functionally versatile phenotypes may allow consumers to specialize on certain resources when preferred resources become scarce (Murdoch, 1969). Examples of weak links between morphological specialization and diet have been found in several ecosystems, including highly diverse coral reefs where most lineages of wrasses and parrotfishes have shown high levels of trophic versatility (Bellwood, Wainwright, Fulton, & Hoey, 2006). This challenges the traditional view that local community structure derives mainly from niche‐partitioning and opens the possibility for alternative hypotheses that metapopulational dynamics (e.g., mass effects) and regional species extinction probabilities (e.g., Lottery Competition and Neutral Theory) are strong determinants of the structure and diversity of local communities (Bell, 2001; Chesson & Warner, 1981; Hubbell, 2001; Sale, 1977). Either way, the relationships between phylogeny, morphological similarity and indicators of ecological performance (e.g., diet) remain poorly documented, this in spite of the fact that numerous studies have assumed morphological traits and/or phylogeny are effective surrogates for species niches when analyzing patterns of community structure (e.g., Cooper, Rodríguez, & Purvis, 2008; Côte, Kuczynski, & Grenouillet, 2019; Kraft, Valencia, & Ackerly, 2008).

**FIGURE 1:**
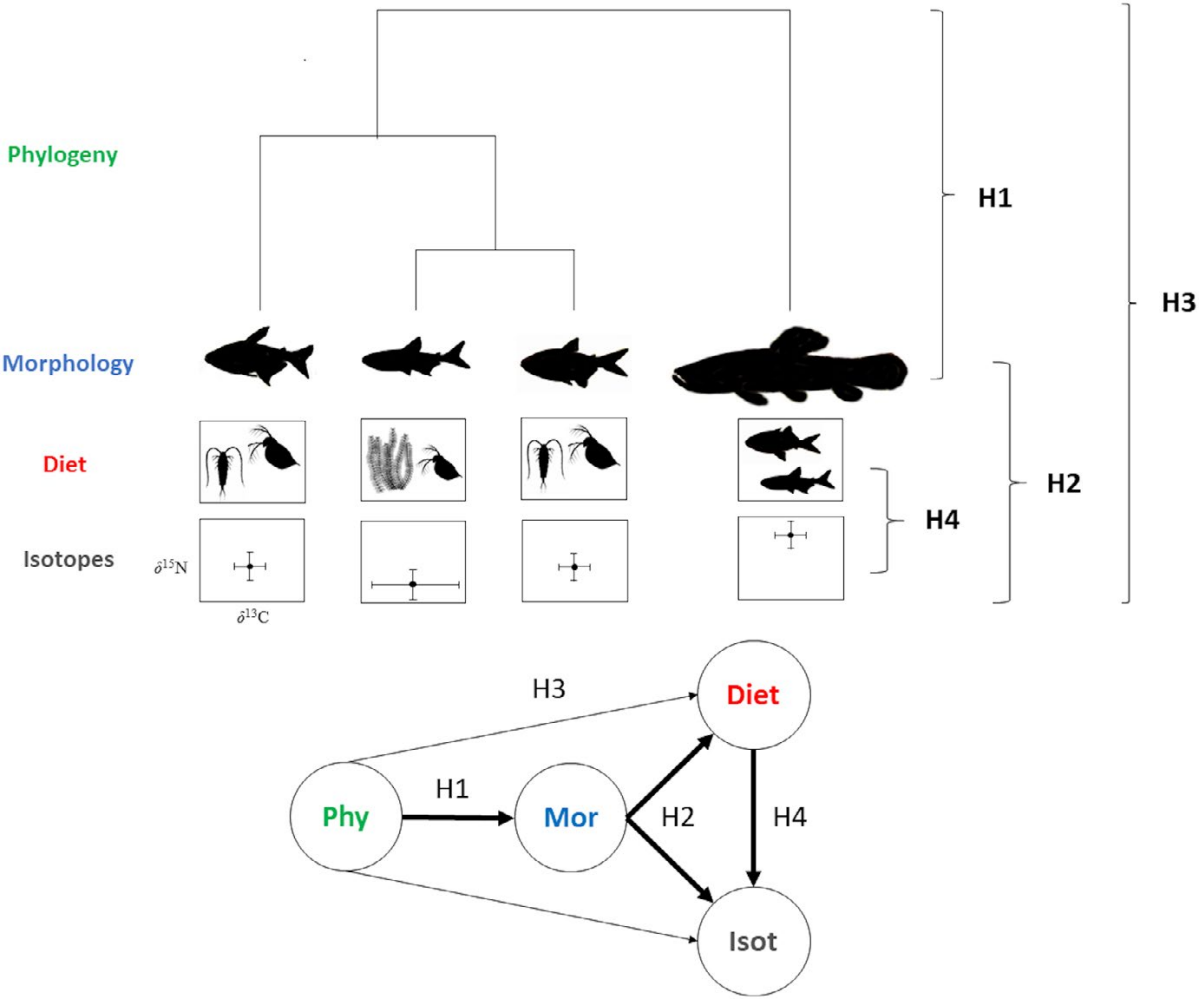
Theoretical model for interrelationships among phylogeny, morphological traits, diet, and isotopic ratios. We hypothesized that: (a) Morphological trait variation has a strong phylogenetic signal (H1); (b) species with similar morphological traits have high diet overlap and similar isotopic ratios (H2); (c) phylogeny influences dietary and isotopic ratios only indirectly and therefore has a weaker relationship with dietary and isotopic patterns than morphological traits (H3); and (d) species with similar isotopic ratios have higher diet overlap (H4)

Here, we investigated species similarity with respect to morphological traits, diet, isotopic ratios, phylogeny, and patterns of intercorrelation among these variables using datasets for freshwater fishes from streams in Central and South America. Fish assemblages in these streams have high taxonomic, morphological, and ecological diversity (Winemiller, 1990). Streams in both regions have seasonal hydrology that causes changes in food resource availability and fish diets (Peterson et al., 2017; Winemiller, 1989, 1990). Previous research revealed significant food resource partitioning throughout the year (Peterson et al., 2017; Winemiller, 1989; Winemiller & Pianka, 1990). What remains unclear is the degree to which morphological traits and phylogeny are associated with patterns of resource partitioning in these diverse fish assemblages. Earlier studies evaluated trophic ecology based on dietary analysis, and here, we analyze those data in conjunction with stable isotope data obtained from some of the same specimens that were preserved and archived in natural history collections. Analysis was restricted to the dry periods and potential differences between sites were considered. Four hypotheses (Figure 1) were tested: (a) Morphological traits have a significant phylogenetic signal; (b) species with similar morphological traits have high dietary overlap and similar isotopic ratios; (c) phylogeny affects diet and isotopic ratios only indirectly and therefore has a weaker association with diet and isotopic ratios than morphological traits; and (d) species with similar isotopic ratios have higher dietary overlap.

## 2 METHODS

### 2.1 Fish samples

**FIGURE 2:**
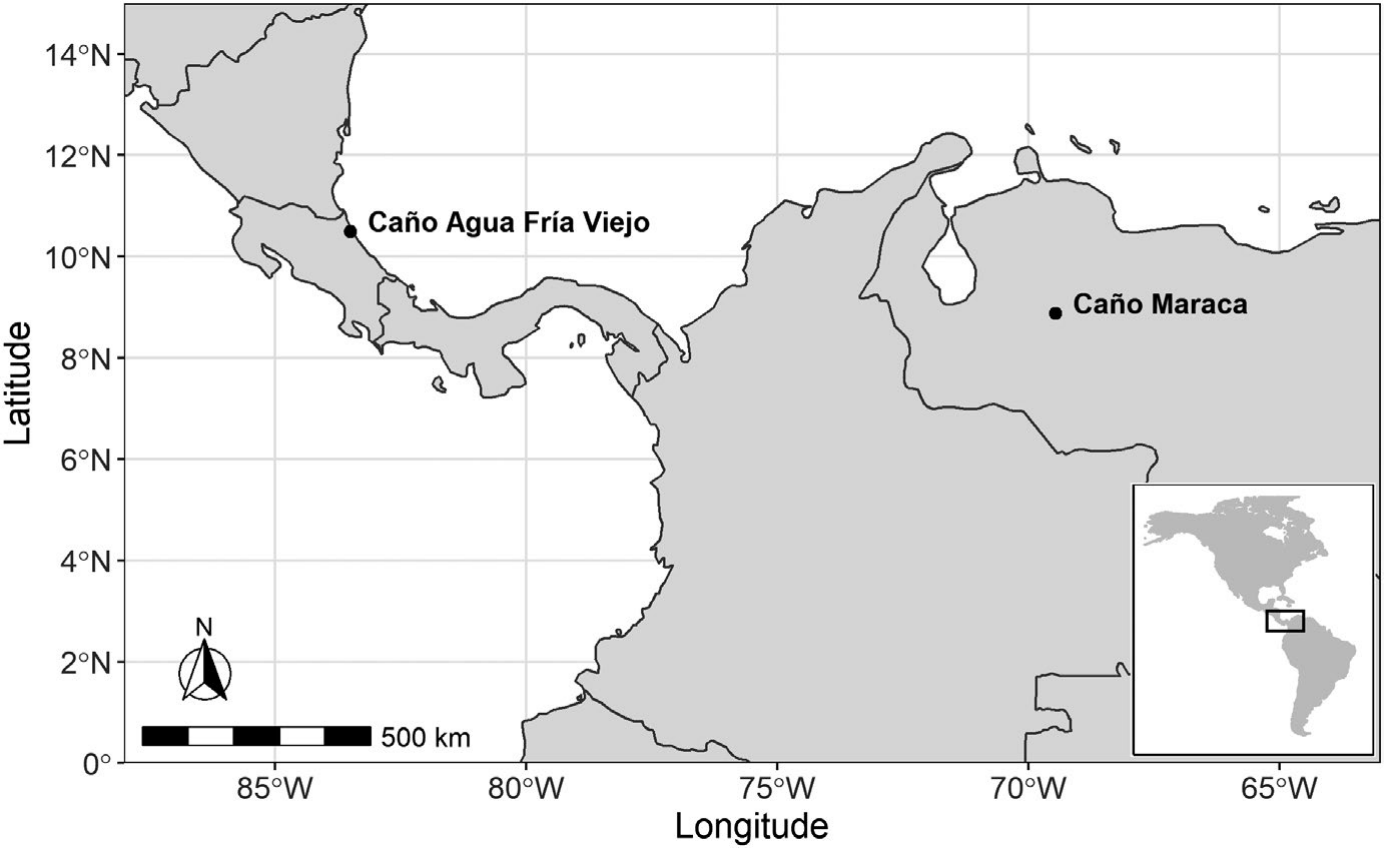
Location of the two streams analyzed in this study. Caño Maraca is a swamp creek situated in the Western Llanos of Venezuela, whereas Caño Agua Fría Viejo is a coastal stream located near the confluence of the Río Tortuguero with the Caribbean Sea in Costa Rica

Fishes were sampled in 1984 and 1985 from Caño Maraca, a swamp creek located in the Western Llanos of Venezuela, and Caño Agua Fría Viejo, a coastal stream located approximately 10 km upstream from the confluence of the Río Tortuguero with the Caribbean Sea in Costa Rica (Figure 2; Winemiller, 1990). At each site, fishes were collected monthly during an entire year using dip nets, gillnets, and seine nets to obtain a reasonably complete sample of the local fish assemblage during each month (Winemiller, 1990). Sampled fish were identified, measured for standard length (SL, mm), and several specimens of the most abundant species were placed in 10% formalin solution for up to 10 months, rinsed, transferred to 70% ethanol solution, and deposited in the Texas Natural History Collection (TNHC) at The University of Texas at Austin.

Here, we restricted our analysis to the most abundant fish species (45 species from Caño Maraca and 24 from Caño Agua Fría Viejo; Table S1) collected only during the dry and transition periods (September to April in Caño Maraca, and March to May and September to October in Caño Agua Fria Viejo). This restriction was necessary to standardize environmental conditions and facilitate comparisons between isotopic ratios and diet data. Moreover, the majority of preserved specimens deposited in the TNHC and available for isotopic analysis (see below) were collected during the dry and transition periods. The number of species included in this study compressed ~54% of the total number of species collected in Caño Maraca (~86% of the total abundance) and ~41% of the species in Caño Agua Fría Viejo (~90% of the total abundance) during an entire annual cycle (Winemiller, 1990). There were not any shared species between Caño Maraca and Caño Agua Fría Viejo in the database analyzed in this study (Table S1).

### 2.2 Phylogenetic data

Phylogenetic relationships were based on a supertree created from analysis of molecular data (multiple genes) from ca. 11,000 fish species (marine and freshwater species) and time‐calibrated using fossil records (Chang, Rabosky, Smith, & Alfaro, 2019; Rabosky et al., 2018). For 21 out of 68 species in our dataset (~31%), genetic information was not available, and those species were not present in the published supertree. We placed those species in positions on the tree occupied by their most closely related taxon, usually a congeneric species (Table S3). We then created a similarity matrix whereby the phylogenetic relationship of each pair of species was expressed as cophenetic distances.

### 2.3 Morphological data

Twenty‐six morphological traits related to feeding, locomotion, and habitat preference (Gatz, 1979; Winemiller, 1991) were measured: standard length, body depth, body width, caudal peduncle length, caudal peduncle height, caudal peduncle width, body depth below midline, head length, head depth, eye position, eye diameter, mouth width, snout length (shut), dorsal fin height, dorsal fin length, pectoral fin length, pectoral fin height, caudal fin length, caudal fin height, pelvic fin length, anal fin length, anal fin height, gut length, mouth orientation (superior, terminal, sub‐terminal, inferior), tooth shape (absent, unicuspid, multicuspid, conical, triangular serrated), and gill raker shape (absent, short/blunt/toothlike, intermediate/long and sparse, long and comb‐like). Measures were taken from 3 to 9 preserved specimens of each species according to methods reported by Winemiller (1991; Table S1). To reduce variation associated with ontogeny, we restricted measurements to the largest specimens available, most of which were classified as adults based on reported sizes at maturation (Winemiller, 1989; Fishbase https://www.fishbase.in/search.php). Following the protocol described by Winemiller (1991), linear measurements of various body and fin dimensions were converted to proportions to control for the effect of body size, and then specimen proportions were averaged for each species. Body size ratios can introduce allometric biases in morphometric analyses (Albrecht, Gelvin, & Hartman, 1993), however, this source of potential bias should have little influence for broad interspecific comparisons (Winemiller, 1991). Besides, body size ratios have straightforward ecological and functional interpretations (Montaña & Winemiller, 2013; Villéger et al., 2017; Winemiller, 1991) and have been widely used in functional ecology studies (e.g., Su et al., 2019; Toussaint et al., 2018). Finally, we constructed a similarity matrix based on Gower distance; this approach was used because the dataset contained three morphological traits (tooth shape, gill raker shape, mouth position) that were categorical.

### 2.4 Isotopic data

Isotopic analysis of δ^13^C and δ^15^N were conducted on large preserved specimens (composed mainly of adults) deposited in the TNHC (see Fish Data section). For most species, we sampled 3 individuals, although in a few cases this number was higher (max = 7) or lower (min = 2) depending on availability of preserved specimens from the field studies (Table S1). Although not uncommon in community ecology studies (e.g., Montaña, Ou, Keppeler, & Winemiller, 2020), small isotopic sample sizes may provide poor representation of species/population isotopic signatures when there is high variation associated with isotopic samples. This could ultimately weaken the association between isotopic ratios and other datasets (e.g., diet and morphological traits). We decided to retain species with small samples for isotopic analysis for two main reasons: (a) Standard deviation around the average values of δ^13^C and δ^15^N was relatively small compared to the average of each species (Figure S1); and (b) removing species with small samples size would reduce the number of species analyzed and, consequently, reduce the representativeness of each community.

At the time of tissue sampling, the deposited species had been preserved for the past 34–35 years. Studies have indicated that the preservation method can affect values of δ^15^N and δ^13^C, but changes seem to be small when compared to natural fractionation processes and are directionally uniform (Arrington & Winemiller, 2002; Edwards, Turner, & Sharp, 2002; Sarakinos et al., 2002). Several studies have performed stable isotope analysis using material from preserved specimens to reconstruct feeding interactions (e.g., Araújo, Bolnick, Martinelli, Giaretta, & Dos Reis, 2009; Kishe‐Machumu, van Rijssel, Poste, Hecky, & Witte, 2017), including some stored as long as the ones used in our study (e.g., English, Green, & Nocera, 2018).

Fish muscle tissue samples were removed from the ventrum just anterior to the anus (the exception was gymnotiforms, for which tissue was taken anywhere from the abdominal region because the anus is located just posterior to the head). Tissue samples were rinsed in distilled water, dried in an oven for 48 hr at 60°C, and then ground to a fine pounder using pestle and mortar. Subsamples weighing 10–30 mg were packed into Ultra‐Pure tin capsules (Costech Analytical, Valencia, California, USA). The encapsulated samples were sent to the Analytical Chemistry Laboratory of the Institute of Ecology at the University of Georgia (USA) for analysis of stable isotope ratios of δ^13^C and δ^15^N. Samples were dry combusted (microDumas technique) using a Carlo Erba CHN elemental analyzer, and the purified gases released from the process were introduced into a Finnigan Delta C mass spectrometer. Stable isotope ratios were quantified as deviations relative to standard materials (atmospheric nitrogen for δ^15^N and Pee Dee Belemnite for δ^13^C). Isotopic ratios had a precision of ≤1.5% for δ^15^N and ≤1% for δ^13^C, measured as the maximum deviation to the mean of bovine (Standard Reference Material [SRM] 1577c) reference samples (measured after every 12 fish tissue samples).

A similarity matrix based on isotopic data was constructed to compare the isotopic ratios of each pair of species. Distance between species was calculated using Euclidean distance after the data were standardized (zero mean and unit variance).

### 2.5 Diet data

Diet analysis was conducted by Winemiller (1990), who dissected 30 specimens of each species from each monthly sample when available. For herbivores and detritivores, the number of dissected specimens was reduced to 20 due to low intraspecific diet variation and the much greater time and effort required to analyze gut contents of these fishes (Winemiller, 1990). Because piscivores typically have a high incidence of empty stomachs (Arrington, Winemiller, Loftus, & Akin, 2002) and their gut contents are processed rapidly when compared to omnivores, herbivores, and detritivores, all available specimens of piscivorous species were dissected. We did not restrict our diet analysis to just the largest specimens that were used for isotopic and morphological analysis because this would have compromised the accuracy of the diet estimates. All else being equal, estimates of diet composition are much more sensitive to sample size than are estimates of morphological dimensions and isotopic composition. Consequently, although average values were similar, the variation in the size of fish examined for diet analysis was a little higher than the variation of those used for isotopic and morphological analysis (Table S1). This source of variation could weaken relationships between morphological traits/isotopic ratios and diet especially if intraspecific dietary and morphological variation increases with size (e.g., Keppeler et al., 2015). However, in a previous study (unpublished), we found that restricting the diet data to only adults versus including a broader range of sizes had minimal effect on correlations between morphological traits and diet data. In this sense, the higher body size variation in the diet dataset likely has minimum influence on the correlations between diet and the other datasets, especially in interspecific comparisons like ours.

The volume of each identifiable food category within the material recovered from each fish stomach was estimated either by water displacement in appropriate‐sized graduated cylinders or, for microscopic items, by estimating the area covered on a slide when viewed under a compound microscope and then scaling the percent coverage estimate according to the total volume of the food mass recovered from the gut (Winemiller, 1990). The volumetric method has been widely applied in diet studies of medium–small size fishes (e.g. Peterson et al., 2017; Silva et al., 2010) and is considered an efficient and practical way to estimate food item importance (Hyslop, 1980). Prey was identified to the lowest taxonomic level that was feasible based on the degree of decomposition and observable characters. In most cases, invertebrates were identified to the family or order level, whereas fish prey varied from species‐level to order or even class due to faster digestion rates. Detritus was classified according to particle size as either fine, coarse, or vegetative detritus (i.e., fragments of dead plant material). Algae were classified according to size (unicellular vs. filamentous) and type (diatoms vs. green and cyanobacteria). Plants were classified according to origin (terrestrial vs. aquatic), tissue (e.g., fruit, seed, and leaf) and, in some cases, taxon (e.g., *Wolffia* sp., *Lemna* sp.). Later, food item volumes were transformed into relative abundances (i.e., standardize to vary from 0 to 1) for each fish individual. For more details about the protocol used for diet analysis, see Winemiller (1990).

Pairwise dietary similarity is strongly influenced by data resolution (Yodzis & Winemiller, 1999). In addition, some kinds of food items are functionally more similar than others, and consequently, some fish trophic guilds (e.g., detritivores) tend to reveal higher dietary overlap than others that display greater niche diversification (e.g., piscivores). We developed a simple new approach that takes into account the nested structure of diet data and generates a single distance value for each pair of species. First, we identified the degree of similarity among food items and created a hierarchical scheme that best describes the data structure (Figure S2, Table S2). The hierarchical structure was organized in 7 different vertical levels forming a pyramid‐like structure (Figure S2, Table S2). Food categories were broad at the top of the pyramid and categories were defined more narrowly at the bottom (Figure S2). There is an inherent tradeoff. As food items are combined into broader categories, resolution becomes poorer but the amount of data available increases; conversely, as the taxonomic resolution increases, some food items are eliminated due to limitations of identification caused by digestion and/or difficulty of identifying diagnostic features of organisms. To avoid major data loss at the bottom of the pyramid, we defined some food categories according to functional categories that were easily identifiable (e.g., detritus, vegetation, seeds; Table S2). However, we removed data for food items that were badly fragmented or digested even though they could be recognized as belonging to a broad category, such as macro‐invertebrates or fish (Table S2).

Second, we created a diet matrix for each hierarchical level of the pyramid‐like structure described above (Table S2, Figure S2). The number of specimens dissected varied greatly among species, from 16 to 396 (Table S1; Total *N* = 7,720). We accounted for these differences by rarefying the number of individuals per species based on the value for the smallest sample (*N* = 16) for each hierarchical level. Then, we averaged the food item ingested among all individuals of each species for each hierarchical level. These matrices with species‐averaged data were then transformed into similarity matrices (Bray‐Curtis dissimilarity; Figure S2). This procedure was conducted 1,000 times and the information documenting each computer loop interaction was saved. For each hierarchical level, the 1,000 similarity matrices were averaged. Finally, we averaged the similarity matrices associated with each hierarchical level, forming a unified similarity matrix that summarizes food overlap between species (Figure S2).

### 2.6 Data analysis

For all datasets, species from all sites were combined into the same similarity matrix as exploratory analysis indicated a low statistical power caused by the small sample size for Caño Agua Fria Viejo (*N* = 24 species). In order to account for potential site differences, we created a binary similarity matrix (herein referred to as site similarity matrix), where 0 and 1 indicate species from the same and different sites, respectively. Then, we conducted partial Mantels to test the correlation between all possible pairings of four similarity matrices that based on different types of data (diet, isotopic ratios, morphological traits, and phylogeny) using the site similarity matrix as a covariable. We also used the Mantel test to investigate the influence of the site similarity matrix on the phylogenetic, morphological, dietary, and isotopic similarity matrices. Mantel and partial Mantels were based on the Spearman correlation statistic which relaxes the assumption of linear relationship assumed by the Pearson statistic (Dietz, 1983; Mantel, 1967). Significance was assessed by permuting the rows and columns of the similarity matrix 10,000 times and comparing the observed value.

To explore the structure of the similarity matrices, dendrograms were created using the UPGMA (unweighted pair group method with arithmetic mean) algorithm. UPGMA method was chosen after comparing results from other cluster methods (Ward D, Ward D2, Single, Complete, WPGMA, WPGMC, UPGMC). This was done by comparing correlation values between the cophenetic distance generated from the dendrogram and the initial distance between the data, i.e. the highest correlation indicates the most representative cladogram of the original similarity matrix (Cachera & Le Loc'h, 2017; Mouchet & Mason, 2008). We created pairwise tanglegrams to compare the similarity between dendrograms of different datasets (e.g., phylogeny, morphological traits). To improve the visualization of the tanglegrams, we used the untangle function (algorithm step2side) of the R package dendextend and colored connecting lines according to taxonomic order and trophic groups. Trophic groups were based on diet data and classified into five groups: Herbivorous/Detritivores (>70% of plant/detritus ingested), omnivores 1 (>30% of plants/detritus and >30% of invertebrates ingested), invertivorous (>70% of invertebrates ingested), omnivores 2 (>30% of invertebrates and >30% of fish ingested), and piscivores (>70% of fish ingested). We also calculated the topological similarity between dendrograms using the score proposed by Nye et al. (2006). The algorithm proposed by Nye et al. (2006) finds the best one‐to‐one mapping of branches among a pair of dendrograms by comparing a calculated similarity score for the clades separated by each branch; the similarity score of the best mapping represents the degree of association between the dendrograms (Nye et al., 2006). The similarity score generated by the algorithm is a measure of the percentage of matched branches between two compared trees and varies from 0 (branches completely unmatched) to 1 (branches completely matched) and is insensitive to the number of terminal nodes. This algorithm has been shown to be superior to other topological similarity metrics, including the ones that take branch length into account, when dendrogram topology is not highly similar (Kuhner & Yamato, 2015). Finally, we calculated the phylogenetic signal associated with each dendrogram (diet, isotopic ratios, morphological traits) using a method similar to the one described by Cachera and Le Loc'h (2017). More specifically, we generated a quantitative state for each tip of each dendrogram using Brownian simulations (value for ancestral state = 0, instantaneous variance = 0.1). We then tested the phylogenetic signal of these quantitative states using Abouheif's *C*_mean_ index (Abouheif, 1999), which performs better than other indexes under the Brownian motion (BM) model of evolution (Münkemüller et al., 2012). This procedure was repeated 10,000 times, generating a distribution of Abouheif's *C*_mean_ index values for each dendrogram. Abouheif's *C*_mean_ varies from −1, when no phylogenetic signal is detected, to 1, when the signal is complete. Because topological similarity and phylogenetic signal of tanglegrams do not control for potential effects of site‐specificity, we also conducted these analyses for each site individually. Besides that, we standardized isotopic ratios (zero mean and unit variance) per site to account for potential differences in δ^13^C and δ^15^N enrichment between sites.

Concerns have been raised regarding the power of distance‐based tests, such as (partial) Mantel, to detect correlations between datasets (Legendre & Fortin, 2010; Legendre, Fortin, & Borcard, 2015). We, therefore, conducted a complementary approach using canonical analyses to confirm results generated by partial Mantels. Canonical analysis has far greater statistical power than distance‐based tests (Legendre & Fortin, 2010), but it requires a reasonable number of sampling units per variable to avoid data overfitting. In our dataset, the number of variables (e.g., 26 morphological traits) was high compared to the number of sampling units (i.e., 65 species). To overcome this limitation, we reduced the dimensionality of the predictor datasets using Principal Coordinates Analysis (PCoA). Phylogeny was considered a predictor of all datasets. Morphological trait data were set as a predictor of both dietary and isotopic data, and dietary data were set as the predictor of the isotopic data. We then selected the most relevant PCoA axes using scree plots. After evaluating scree plots for gradients produced from PCoA performed separately for phylogenetic, morphological, and dietary similarity matrices, we selected 4 axes to describe phylogenetic relationships (cumulative variation explained = 94%), 14 axes for morphological traits (78%), and 10 axes for diet (63.4%).

Axes of each dataset were then correlated with the response data using Redundancy Analysis (RDA; isotopic ratios modeled by morphological traits, phylogeny, and diet) and Distance‐Based Redundancy Analysis (db‐RDA; diet modeled by morphological traits and phylogeny, and morphological traits modeled by phylogeny). In each case, we simplified the canonical models via forward selection based on permutation tests (10,000 randomizations) to include only significant explanatory variables (i.e., PCoA axes) in the models. Forward selection retained all four of the dominant phylogenetic axes for the phylogeny‐morphological traits comparison; three phylogenetic axes for the phylogeny‐diet comparison; the first two phylogenetic axes for the comparison of the phylogeny‐isotopic ratios; six PCoA axes for the morphological traits‐diet comparison; three PCoA axes for the morphological traits‐isotopic ratios comparison; and five PCoA axes for the diet‐isotopic ratios comparison.

After defining the best model for each comparison, we assessed the unique contribution of the explanatory dataset to a given response dataset by conditioning its effect by site (Caño Maraca or Caño Agua Fría Viejo; for more details about the method used, see Peres‐Neto et al., 2006). The significance of these contributions was assessed using an ANOVA‐like permutation test for canonical analysis (Legendre & Legendre, 2012). Finally, we used the first two PCoA axes from the morphological data and the phylogenetic tree to construct a phylomorphospace plot, which is a projection of the phylogenetic tree into morphospace (represented by the first two PcoA axes). The tips of the tree were colored according to the species trophic groups, *δ* ^15^N, and *δ* ^13^C values to better visualize the link between phylogeny, morphology, and trophic ecology.

All analyses were conducted in R (R Core Team, 2019). Canonical analysis and (partial) Mantel tests were carried out in vegan (Oksanen et al., 2019). Dendrograms, tanglegrams, and Nye's topological comparisons were conducted in the packages stats (R Core Team, 2019), dendextend (Galili, 2015) and TreeSearch (Smith, 2018), respectively. Brownian motion simulations and phylomorphospace plots were carried in the package phytools (Revell, 2012), and the Abouheif's *C*_mean_ index was calculated in the package adephylo (Jombart, Balloux, & Dray, 2010). Fish phylogeny was retrieved from the fishtree package (Chang et al., 2019).

## 3 RESULTS

### 3.1 Morphology–phylogeny association

**FIGURE 3:**
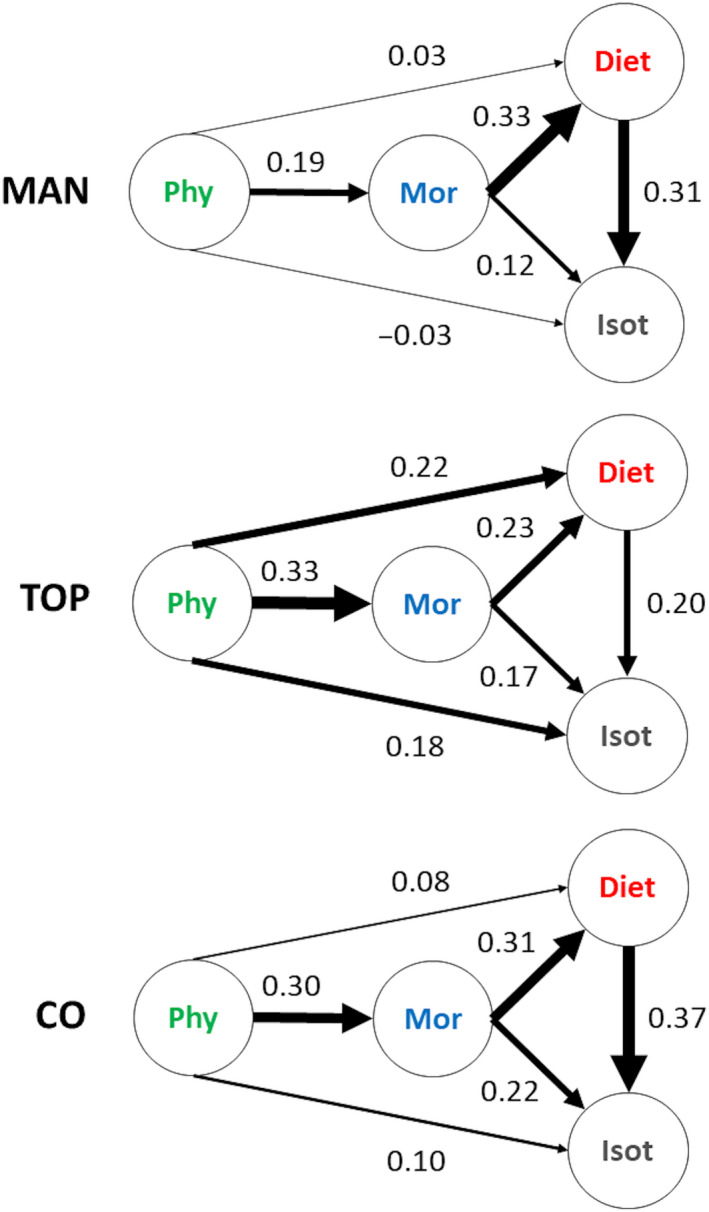
Pairwise relationships between phylogeny (Phy), morphological traits (Morpho), isotopic ratios (Isot), and diet based on three different approaches: partial Mantel (MAN), topological similarity (TOPO), and constrained ordinations (CO). Sites were used as covariables for partial Mantel and constrained ordinations, but not for topological similarities (topological similarities calculated for each site individually can be found in Table 1). Arrows thickness is proportional to the association among datasets, which was assessed using Spearman's statistic in the partial Mantels, topological similarity based on the algorithm proposed by Nye et al. (2006), and partial *R* ^2^ for constrained ordinations (RDA). The actual values of these statistics are also presented associated with each connecting arrow

**FIGURE 4:**
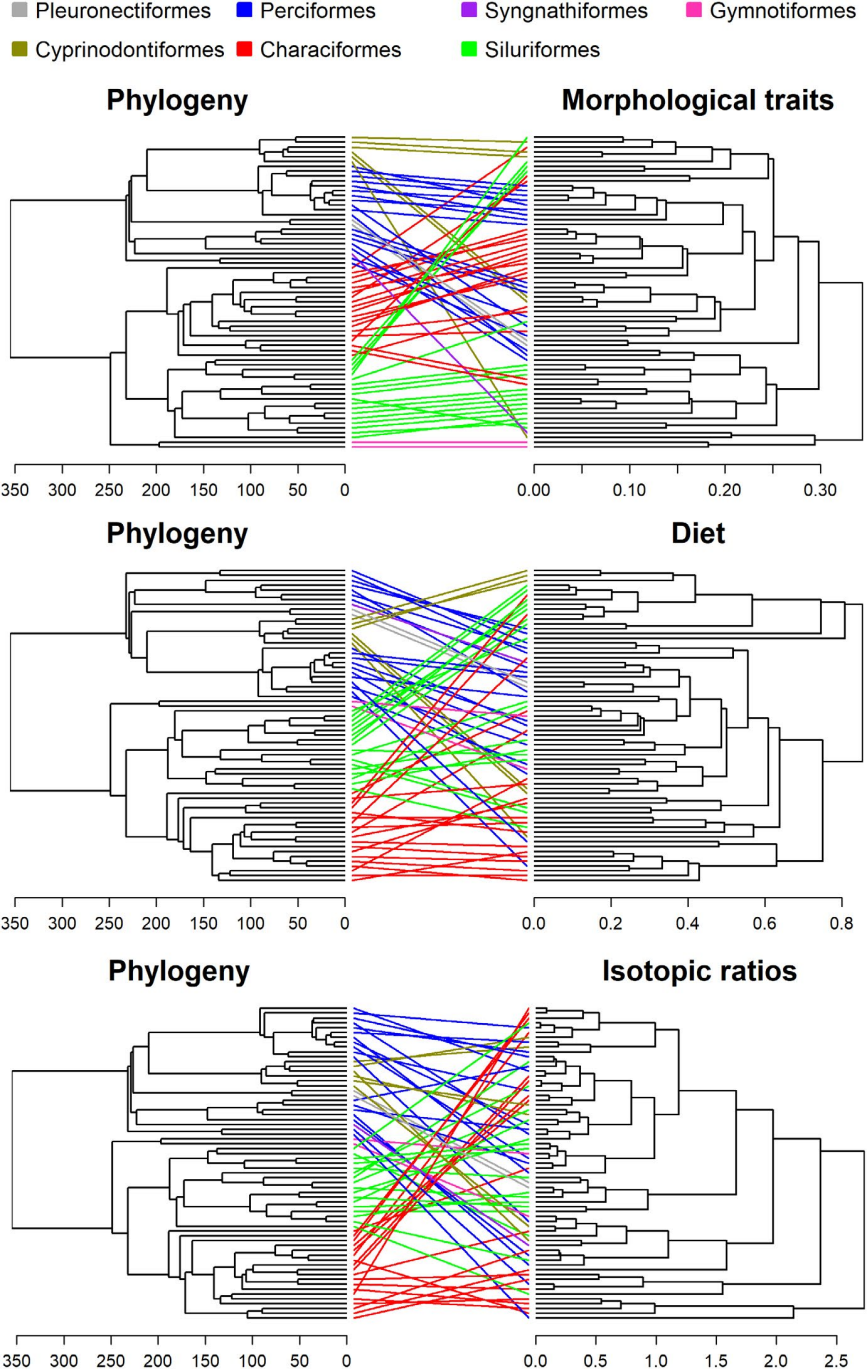
Tanglegrams constructed for pairwise comparisons between phylogeny and morphological traits, phylogeny and diet, and phylogeny and isotopic ratios dendrograms. Dendograms were constructed using the UPGMA algorithm and using species of all sites combined. We used an untangle function (algorithm step2side) to improve the visualization of the tanglegrams. Colors represent different taxonomic orders. Tanglegrams constructed for each site separately can be found in Figure S3

**FIGURE 5:**
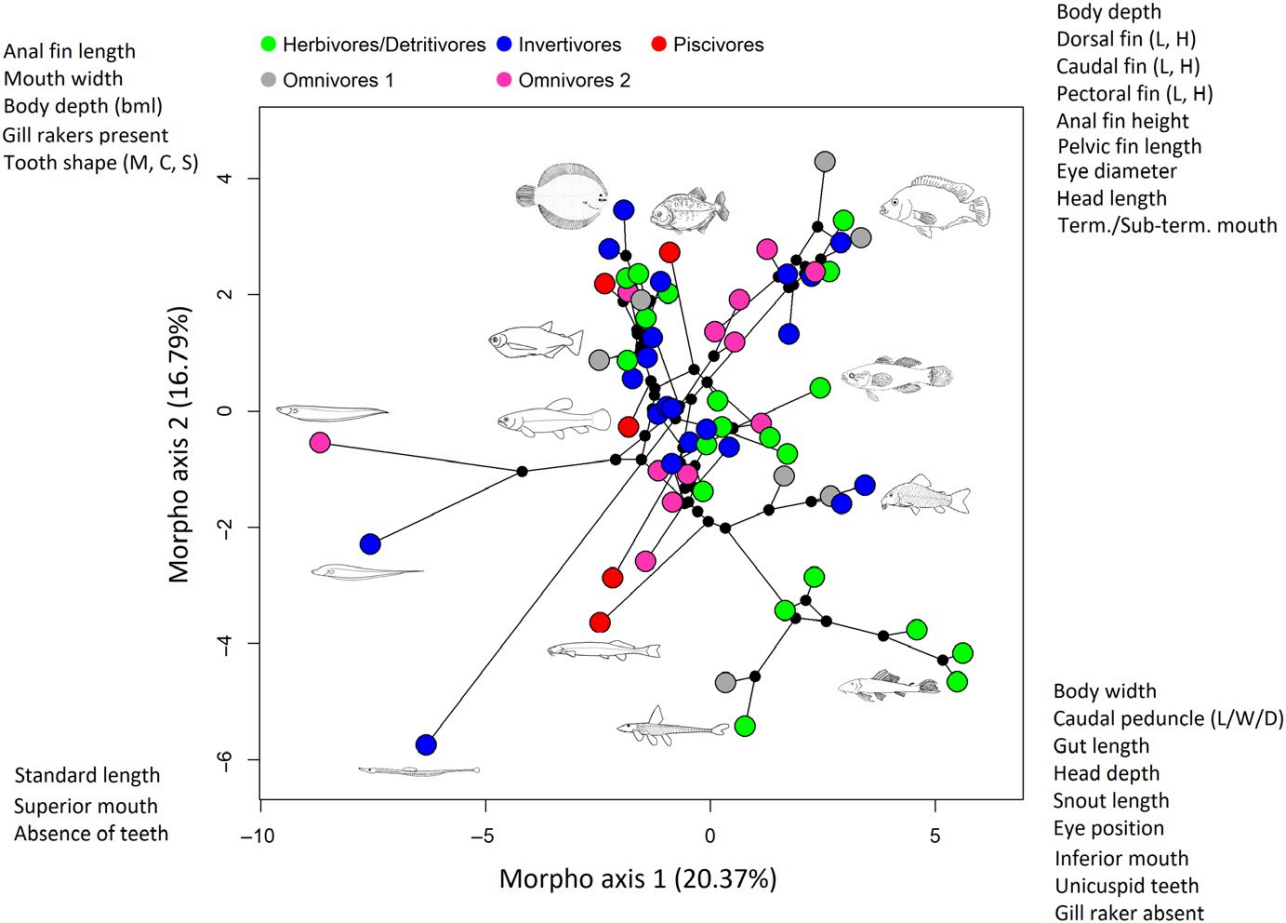
Projection of the phylogenetic tree into morphospace, which is represented by the first two axes of the Principal Coordinate Analysis (PCoA). Tree internal nodes are represented by small black dots. Tips of the tree are colored according to species trophic group based on diet analysis. Morphological traits associated with each side of the morphological space are also shown. L = Length, W = Width, D = Depth, H = Height, bml = Below middle line, M = Multicuspid, C = Conical, S = Serrated triangular

Partial Mantel results indicated that the phylogenic similarity matrix was significantly associated with the morphological similarity matrix (*r* = .19, *p* < .001; Figure 3). Dendrograms based on phylogenetic and morphological data had the highest levels of topological similarity and phylogenetic signal (Tables 1 and 2). The partial db‐RDA (conditioned by sites) also confirmed that phylogeny significantly influenced fish morphological traits (*F*_4,60_ = 6.07, *p* < .001, Adj. *R* ^2^ = .30; Figure 3). Tanglagrams and phylomorphospace plots indicated that morphological traits are particularly conserved in Gymnotiformes (knifefishes) and Pleuronectiformes (flatfish) (Figure 4) with species presenting a distinct eel‐like body shape with a long anal fin in the former and a flat body with strong asymmetry in the latter (Figure 5). A large proportion of Siluriformes (catfishes), Perciformes (perch‐like fishes), and Characiformes (characins and their allies) also had relevant conservation of traits. Siluriformes, particularly loricariids and callichthyids (armored catfishes), were mainly associated with morphological adaptations to inhabit benthic environments (e.g., inferior mouths and depressed body shape) and feed on attached algae and detritus (long guts and unicuspid teeth) (Figures 4 and 5). Perciformes, particularly cichlids, were characterized by deep body shapes with conspicuous fins (Figure 5). Characiformes, particularly the family Characidae, were mainly associated with fusiform body shapes and terminal mouths, typical of pelagic fishes, and multicuspid teeth (Figure 5).

**TABLE 1:** Topological similarity of the phylogenetic, morphological, dietary, and isotopic dendrograms

Comparison	All	Maraca	Agua Fria
Phy versus Traits	0.33	0.24	0.11
Phy versus Diet	0.22	0.13	0.09
Phy versus Iso	0.18	0.12	0.07
Traits versus Diet	0.23	0.15	0.10
Traits versus Iso	0.17	0.13	0.09
Diet versus Iso	0.20	0.13	0.09

Note

Dendrograms were created for all species combined (ALL), and also for species in each site individually (Caño Maraca and Caño Agua Fria Viejo). Topological similarity was calculated according to the algorithm proposed by Nye et al. (2006) and each value is given in percentage (higher values indicate higher similarity). Phy = Phylogeny, Traits = Morphological traits, Iso = Stable isotope ratios. A comparison between the results of topological similarity, partial Mantel tests, and constrained ordination methods can be found in Figure 3.

**TABLE 2:** Average phylogenetic signal associated with dendrograms created from morphological (traits), dietary, and isotopic datasets

Dendrogram	All	Maraca	Agua Fria
Morphological traits	0.22 (−0.02, 0.47)	0.25 (−0.05, 0.56)	0.11 (−0.20, 0.45)
Diet	0.13 (−0.09, 0.38)	0.11 (−0.15, 0.43)	0.02 (−0.25, 0.33)
Isotopic ratios	0.04 (−0.14, 0.24)	0.03 (−0.18, 0.28)	−0.05 (−0.28, 0.22)

Note

Dendrograms were created for all species combined (ALL) and for species from a single site (Caño Maraca, Caño Agua Fria Viejo). The procedure used here is similar to the one described by Cachera and Le Loc'h (2017), where a quantitative state for each tip of each dendrogram is created using Brownian simulations (value for ancestral state = 0, instantaneous variance = 0.1). We used the Abouheif's *C*_mean_ index (Abouheif, 1999) as our measured of phylogenetic signal. Abouheif's *C*_mean_ varies from −1, when no phylogenetic signal is detected, to 1, when the signal is complete. Values in parentheses are the 2.5% and 97.5% quantiles based on the variation associated with the Brownian simulations (10,000 times for each dendrogram). The distribution of Abouheif's *C*_mean_ values can be found in Figure S4.

### 3.2 Association of phylogeny with diet and isotopes

**FIGURE 6:**
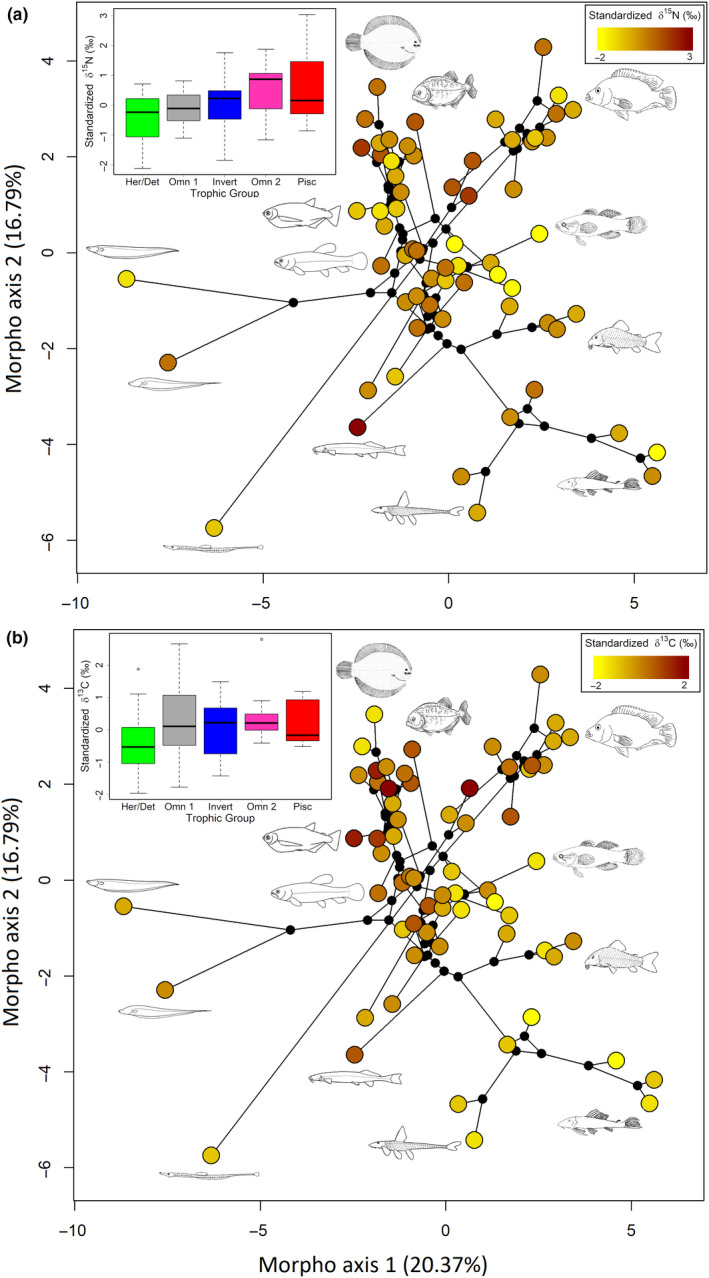
Projection of the phylogenetic tree into morphospace, which is represented by the first two axes of the Principal Coordinate Analysis (PCoA). Tree internal nodes are represented by small black dots. Tips of the tree (species) are colored according to its signature of either *δ* ^15^N (a) or *δ* ^13^C (b). Box plots showing variation of *δ* ^15^N (a) and *δ* ^13^C (b) across different trophic groups can be found in the superior left corner of each panel

According to partial Mantel analysis, the phylogenetic similarity matrix was not associated with neither the diet (*r* = .03, *p* = .12, Figure 3) nor the isotopic similarity matrix (*r *= −.03, *p* = .84, Figure 3). Phylogenetic dendrograms had intermediate scores for topological similarity with diet dendrograms and only low scores with isotopic dendrograms (Table 1). Despite some degree of overlap, the phylogenetic signal of the diet dendrogram was also weaker than the phylogenic signal of morphology, but stronger than the isotopic dendrogram (Table 2). The constrained ordinations also indicated that despite being significant (Phylogeny versus Diet: *F*_2,62_ = 6.07, *p* = .002, Adj. *R* ^2^ = .08; Phylogeny versus Isotopes: *F*_2,62_ = 4.63, *p* = .005, Adj. *R* ^2^ = .10), the association of phylogeny with diet and isotopic ratios were weaker than the association between phylogeny and morphological traits (Figure 3). Although some clades were consistently composed of the same trophic groups ( e.g., loricariids [Siluriformes] were mainly herbivores/detritivorous), most clades had species with multiple feeding strategies (Figures 4 and 5). No strong gradient of neither *δ* ^13^C nor *δ* ^15^N were found along fish phylogeny, indicating that these elements are not effective to distinguished between different phylogenetic clades (Figure 6).

### 3.3 Association of morphology with diet and isotopes

**FIGURE 7:**
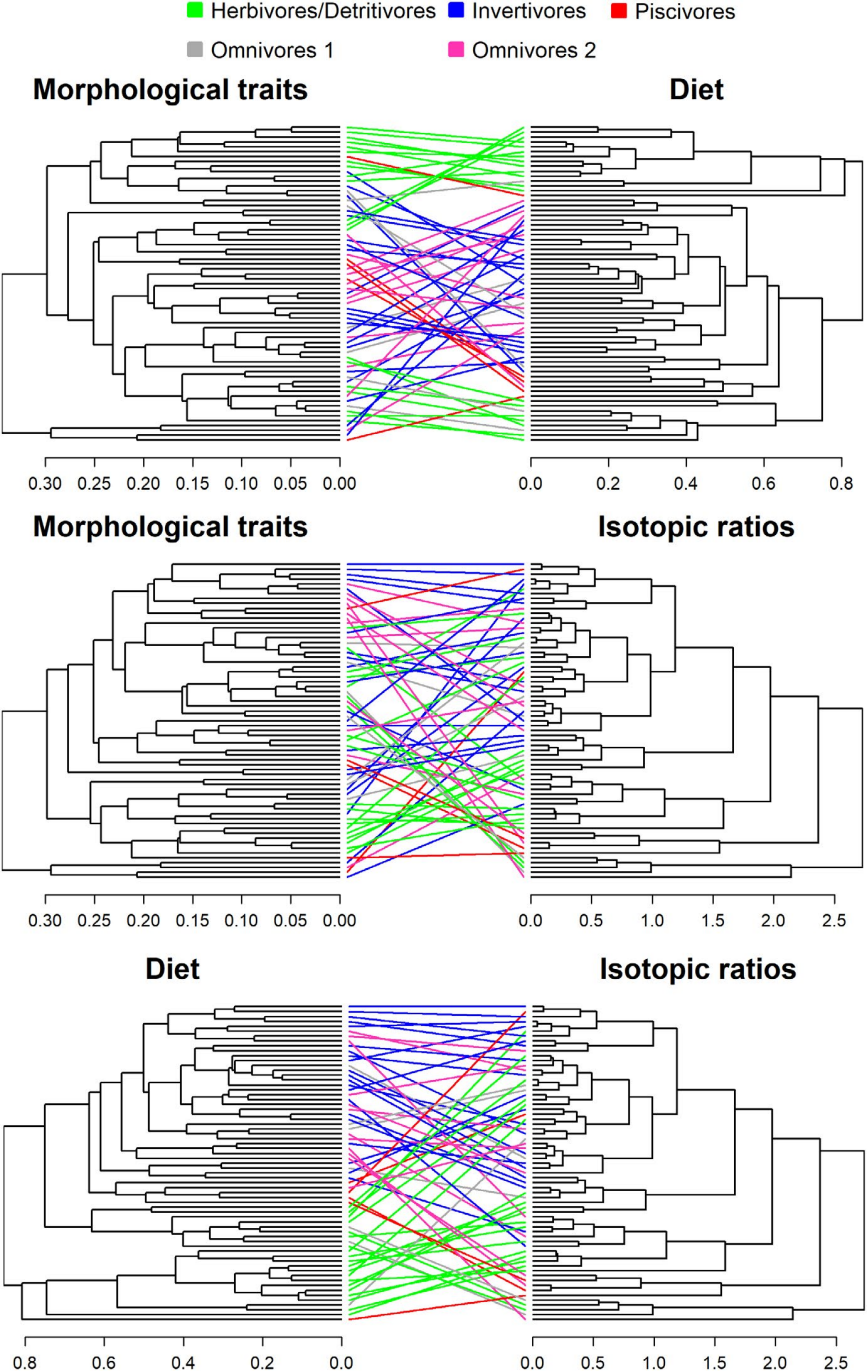
Tanglegrams constructed for pairwise comparisons between morphological traits and diet, morphological traits and isotopic ratios, and diet and isotopic ratios dendrograms. Dendograms were constructured using the UPGMA algorithm and using species of all sites combined. We used an untangle function (algorithm step2side) to improve the visualization of the tanglegrams. Colors represent different trophic groups. Tanglegrams constructed for each site separately can be found in Figure S3

The partial Mantel results indicate that morphological similarity matrix was significantly associated with both diet (*r* = .33, *p* = .001, Figure 3) and isotopic similarity matrices (*r* = .12, *p* = .03, Figure 3), although the relationship was weaker for the latter. The morphological dendrogram had an intermediate score (0.22) for topological similarity with diet dendrograms (Table 1; Figure 3). This similarity score was a little stronger than the similarity score found between the morphological dendrogram and isotopic dendrograms (0.17; Table 1; Figure 3). Constrained ordinations also confirmed a significance association of morphological traits with diet (*F*_6,58_ = 5.72, *p* < .001, Adj. *R* ^2^ = .31) and isotopic signatures (*F*_3,61_ = 7.04, *p* < .001, Adj. *R* ^2^ = .22); the latter being stronger than the former (Figure 3). Associations between morphology and diet varied between trophic groups, being typically stronger for herbivores/detritivores (Figures 5 and 7). However, the same pattern did not hold for the relationship between morphology and isotopic ratios, which was noisier (Figures 6 and 7).

### 3.4 Diet and isotopes association

According to the partial Mantel, diet and isotopic similarity matrices were significantly correlated (*r* = .31, *p* = .001, Figure 3). Partial RDA analysis also indicated that the diet and isotope relationship was significant (*F*_5,59_ = 8.06, *p* < .001, Adj. *R* ^2^ = .37) and the strongest association among the datasets (Figure 3). Isotopic dendrograms and diet dendrograms had intermediate topological similarity scores (except for the analysis that included only species from Caño Agua Fría Viejo, which had low scores; Table 1, Figure 7). *δ* ^15^N was mainly associated with trophic level, being lower for herbivores/detritivores and higher for omnivores 2 and piscivores (Figure 6). *δ* ^13^C was lower for herbivores/detritivores when compared to all other trophic groups (Figure 6).

### 3.5 Sites differences

Site similarity was moderately associated with phylogenetic relatedness (*r* = .39, *p* < .001), weakly associated with diet (*r* = .07, *p* = .03) and isotopic ratios similarity (*r* = .07, *p* = .04), and not significantly associated with morphological traits similarity (*r* = .02, *p* = .26). Overall, topological similarity and phylogenetic signal conducted for each site individually generated similar patterns (e.g., phylogenetic signal was stronger in morphological traits than in diet and isotopic ratios) compared to the results of analysis conducted with both sites together (Tables 1 and 2; Figure S3). However, the magnitude of correlations values and phylogenetic signals was typically higher in Caño Maraca than in Caño Água Fria Viejo (Tables 1 and 2).

## 4 DISCUSSION

Morphological traits of both tropical freshwater fish assemblages revealed a significant phylogenetic signal, corroborating our first hypothesis. Both phylogeny and morphological traits were associated with fish diets and isotopic ratios; however, morphological traits were stronger predictors of dietary and isotopic ratios than phylogenetic relationships, corroborating our second and third hypotheses. Diet and isotopic ratios were significantly correlated, indicating that species with similar isotopic ratios tend to have relatively high dietary overlap (hypothesis 4). Together, these findings lend some support for approaches in community ecology that rely on species traits to infer niche relationships (e.g., Côte et al., 2019; Kraft et al., 2008). However, high levels of unexplained variation in dietary and stable isotopic ratio data (>60%) suggest caution is warranted when interpreting patterns of community structure based on phylogenetic and morphological data. Although part of this variation may be caused by our methodology (e.g., body and sample size differences between datasets, preservation effects on isotopic ratios), morphology and phylogeny nonetheless may provide a blurred view of species niche relationships. This imprecision could limit their usefulness as proxies in certain kinds of studies that require high precision, such as those aiming to distinguish community assembly processes (Gerhold, Cahill, Winter, Bartish, & Prinzing, 2015); however, morphological traits and phylogenetic relationships should be useful in macroecological studies, such as those exploring trophic diversification (López‐Fernández, Winemiller, Montaña, & Honeycutt, 2012; Winemiller et al., 1995).

The significant phylogenetic signal for morphological traits indicates that closely related species are more morphologically similar than expected at random (Blomberg & Garland, 2002). Associations between phylogeny and morphological traits are expected under a random walk model of evolution (i.e., Brownian motion) that assumes changes are gradual and random due to either genetic drift or random fluctuations in natural selection (Losos, 2008). Other factors, including strong stabilizing selection and genetic constraints associated with pleiotropy, could promote conservatism in trait evolution (Wiens & Graham, 2005). However, we highlight that our method for assessing phylogenetic signal integrated multiple morphological traits, some of which could have evolved in response to different sources of selection (Cadotte, Davies, & Peres‐Neto, 2017). Patterns of evolution for multiple‐traits are often well described by Brownian motion models (Cadotte et al., 2017; Cooper & Purvis, 2010), and, therefore, phylogenetic relationships could be particularly useful to predict general patterns of ecological similarity and function among species assemblages or taxa (Cavender‐Bares, Kozak, Fine, & Kembel, 2009; Mouquet et al., 2012).

Predicting community processes based on functional traits has been considered the “Holy Grail” in ecology (Lavorel & Garnier, 2002). Our results indicated that morphological traits known to influence swimming and feeding performance can serve as proxies for the trophic ecology of freshwater fish. However, morphological traits only explained a moderate fraction of fish dietary variation (coefficient of determination and correlations between 0.12 and 0.33, depending on the method used). Multiple morphological traits may have redundant functions for feeding (Wainwright et al., 2005; Zelditch et al., 2017), which would explain the limited predictive power of morphological traits in our study. Although not investigated in this study, individual feeding also could have influenced dietary patterns among species in our study and reduced the importance of species‐averaged morphology as a valid proxy for trophic interactions. Specialized feeding by individuals has been shown in several fish species, but little is known about intraspecific variation in trophic niches (Bolnick et al., 2003, 2011). Moreover, many fish species have broad diets and display high levels of omnivory (Winemiller, 1990). Predator switching and broad trophic niches may be common in seasonal ecosystems that experience major fluctuations in abiotic conditions and resources availability (McMeans, Mccann, Humphries, Rooney, & Fisk, 2015). Here, we restricted our analysis to periods when water levels were low (dry and transition periods), fish densities were high, aquatic resources were depleted, and diet breadth and interspecific diet overlap tended to be low (Peterson et al., 2017; Winemiller, 1989). Therefore, it is likely that the correlation between morphological traits and diets might have been even lower if we had considered a longer time interval that included all phases of the tropical hydrologic cycle. In this sense, inferences about species niches based only on morphological traits should be made with caution, with the acknowledgment that ecological performance depends on environmental conditions that vary in space and time (Cadotte, Carboni, Si, & Tatsumi, 2019; Kraft, Godoy, & Levine, 2015).

Diet was more strongly correlated with morphological traits than phylogeny, suggesting that morphological traits are the better predictor of trophic interactions. Indeed, partial Mantel analysis even indicated that the association of phylogeny with diets and isotopic ratios was not significant. Morphological traits are usually a stronger predictor of diet data than phylogeny because selective pressures can drive species from different clades to converge on similar phenotypes, allowing them to exploit similar resources (Grant, Grant, Markert, Keller, & Petren, 2004; Winemiller et al., 2015). Conversely, closely related species may undergo character displacement as a result of interspecific competition within areas of sympatry, or they may adapt to exploit different resources under different environmental conditions (Brown & Wilson, 1956; Schluter, 2000). Trophic diversification is observed in many freshwater fish families. For example, some Neotropical cichlids are specialized piscivores (*Cichla* spp.), others invertivores (*Aequidens* spp., *Geophagus* spp.), and others herbivores (*Uaru amphiacanthoides*). Interestingly, herbivory and detritivory, which require specialized gut morphology and physiology (e.g., long guts for longer passage time and enhanced nutritional absorption; Horn, 1989), occur in fishes from very different lineages, including poecilids, loricariids, callichthyids, prochilodontids, and curimatids. The weak association between phylogeny and diet might derive, in part, from the inclusion of neutral genetic sequences unrelated to natural selection during phylogenetic tree construction (Cadotte et al., 2019). In any event, our results suggest that conclusions about species niches and community functional structure based on phylogenetic relationships alone can be misleading, a position argued by others (Gerhold et al., 2015; Mayfield & Levine, 2010).

Diet was significantly correlated with stable isotope ratios, likely reflecting differences in δ^13^C of basal resources in food chains supporting consumers with various trophic niches, and trophic fractionation of δ^15^N indicating vertical trophic positions (Fry, 2006; Layman et al., 2012). The correlation between fish diet and stable isotope ratios was stronger than between morphological traits and isotopic ratios or between phylogeny and isotopic ratios. This indicates that error associated with inferred trophic isotopic enrichment, environmental influences on isotopic signatures of basal sources, effects of body size and metabolism on consumer isotopic ratios, and other factors is not large enough to completely degrade the signal revealing community trophic structure provided by isotopic ratios. On the other hand, morphology and phylogeny were usually more related to diet data than with isotopic ratios, probably reflecting the indirect relationship among trophic niche and stable isotopes (Caut et al., 2009). For example, many loricariid catfishes (Siluriformes) have diets dominated by detritus, algae, and micro‐invertebrates, whereas soles (Pleuronectiformes: Achiridae) feed on both micro‐ and macro‐invertebrates. These dietary patterns were completely lost in dendrograms based on stable isotope ratios. Without knowing the isotopic ratios of the basal resources, it is difficult to determine why some species associations were lost. The use of stable isotope ratios to infer trophic relationships can lead to misleading conclusions if variation in isotopic ratios of the basal resources in food chains supporting consumer biomass is not taken into account (Hoeinghaus & Zeug, 2008; Layman et al., 2012). In this sense, stable isotope ratios should be considered a complement rather than a substitute for diet data (Davis et al., 2012). Stable isotope ratios have the advantage of integrating assimilation of consumed items over time (i.e., several weeks to months depending on tissue type), provided that food resources have sufficiently distinct isotopic ratios (Layman et al., 2012). Dietary analysis provides much greater resolution of trophic niches, but the method merely provides a snapshot of items ingested prior to the organism's capture (e.g., Winemiller, 1990). Combined analysis of dietary and isotopic data can better reveal trophic patterns at the level of the individual organism, community, or taxon (Costa‐Pereira, Rudolf, Souza, & Araújo, 2018).

Stable isotope ratios analyzed in our study were obtained from preserved specimens collected more than three decades ago. Prior research has shown that δ^15^N tends to be slightly elevated and δ^13^C is slightly lower in fish muscle tissue following fixation in formalin and storage in ethanol (Arrington & Winemiller, 2002; Edwards et al., 2002; Kishe‐Machumu et al., 2017; Sarakinos et al., 2002). Given the relatively minor and consistent isotopic changes observed for preserved fish tissues, it has been proposed that archived specimens can provide a reliable data source for isotopic analysis aimed at revealing long‐term trends (Edwards et al., 2002; Sarakinos et al., 2002). We found strong correlations between isotopic and dietary data, moderate correlations between isotopic and morphological data, and weak correlations between isotopic and phylogenetic data. These results further emphasize the importance of scientific collections for food web research. Millions of preserved specimens are housed in natural history collections worldwide, and these could be used to address many ecological questions using stable isotope analysis (Meineke, Davies, Daru, & Davis, 2019). This archived material could advance research on topics ranging from food web ecology to community trophic structure and long‐term changes associated with environmental impacts and climate change (Kishe‐Machumu et al., 2017; Sarakinos et al., 2002; Schmitt et al., 2018).

Species composition and their respective phylogenetic lineages differed between the studied sites. This mainly reflects the isolation and the geological history of the regions where Caño Maraca and Caño Agua Fría Viejo are located (South America and Central America, respectively). Not surprisingly, our analysis indicated that diets and isotopic signatures varied between sites. Phylogenetic signals and patterns of correlation between the different datasets (e.g., diet, morphology) were similar between sites, but values were usually lower for Caño Agua Fría Viejo when compared to analyses conducted for Caño Maraca or with both sites together. These differences could be caused by the low statistical power of our analysis due to a small sample size for Caño Agua Fria Viejo. Because it had more species, Caño Maraca might have contributed more to phylogenetic signal and topological similarity when both sites together. Alternatively, differences in the degree of longitudinal hydrological connectivity and dispersal or environmental fluctuation could have contributed to lower correlations and phylogenetic signal for Caño Agua Fria Viejo. Caño Agua Fría Viejo is located about 10 km from the Caribbean Sea (Winemiller, 1990), and its fish assemblage is open to invasion by estuarine and marine species. Caño Maraca is located further inland within the Orinoco Basin and has more extreme hydrological variation that creates harsh environmental conditions during the dry season (Winemiller, 1990). Seasonal reduction in habitat availability and quality generally reduces niche breadth and interspecific diet overlap (Peterson et al., 2017; Winemiller, 1989; Winemiller & Pianka, 1990), which might strengthen the association between morphology and diet. To investigate these possibilities, future studies exploring relationships between phylogeny, morphological traits, and trophic niche should include more locations and zoogeographic regions that span gradients fluvial connectivity and environmental conditions, including temporal variation.

Ecologists frequently use either phylogenetic or morphological similarity as a proxy for ecological similarity (Morales‐Castilla, Matias, Gravel, & Araújo, 2015). However, few studies have tested these assumed relationships, and this may be due to the great effort required to obtain sufficient empirical data for large numbers of species (Silva et al., 2019). Our analysis of phylogenetic, morphological, dietary, and isotopic data for diverse tropical fish assemblages showed that morphological traits had moderate correlations with diet and weak correlations with stable isotope ratios, whereas phylogeny had weak correlations with both dietary and isotopic data. With recent advances in genomics, phylogenetics, and functional morphology as well as the compilation of associated data into public digital databases, phylogenetic, and functional trait data are becoming more easily available. Despite these advantages, there are important factors that limit the use of phylogeny and morphological traits to infer niche relationships (Cadotte et al., 2017, 2019; Funk et al., 2017; Gerhold et al., 2015). To enhance understanding of community assembly and ecological diversification, future research should further explore methods that integrate phylogeny, morphology, and chemical tracers (e.g., bulk stable isotope ratios, amino‐acid‐specific stable isotope ratios, fatty acid signatures) for analysis of trophic ecology.

## CONFLICT OF INTEREST

None declared.

## AUTHOR CONTRIBUTIONS

**Friedrich W. Keppeler:** Conceptualization (equal); Data curation (supporting); Formal analysis (lead); Funding acquisition (supporting); Investigation (equal); Methodology (lead); Resources (equal); Software (lead); Validation (equal); Visualization (lead); Writing‐original draft (lead); Writing‐review & editing (equal). **Kirk O. Winemiller:** Conceptualization (equal); Data curation (lead); Formal analysis (supporting); Funding acquisition (lead); Investigation (equal); Methodology (supporting); Project administration (lead); Resources (supporting); Software (supporting); Supervision (lead); Validation (equal); Visualization (supporting); Writing‐original draft (supporting); Writing‐review & editing (equal).

## Supporting information

Fig S1

Fig S2

Fig S3

Fig S4

Table S1

Table S2

Table S3

## Data Availability

Phylogenetic data available for download from the R package fishtree (Chang et al., 2019) and the website https://fishtreeoflife.org/ (accessed on 10 November 2019). Average and standard deviation of isotopic ratios for each species can be found in the supplementary materials (Figure S1). Similarity matrices associated with each dataset (phylogeny, morphological traits, diet, and isotopic ratios) were archived in Dryad (https://doi.org/10.5061/dryad.0k6djh9x2).
