# Supplementary material for: Can ancestry and morphology be used as surrogates for species niche relationships?
Source: Ecol Evol. 2020 Jun 3;10(13):6562–78. doi: 10.1002/ece3.6390 (PMC7381567; doi:10.1002/ece3.6390)

Figure S1. Average and standard deviation of δ^15^N (A, C) and δ^13^C (B, D) for each species in each site (Caño Maraca: A, B; Caño Agua Fría Viejo: C, D).


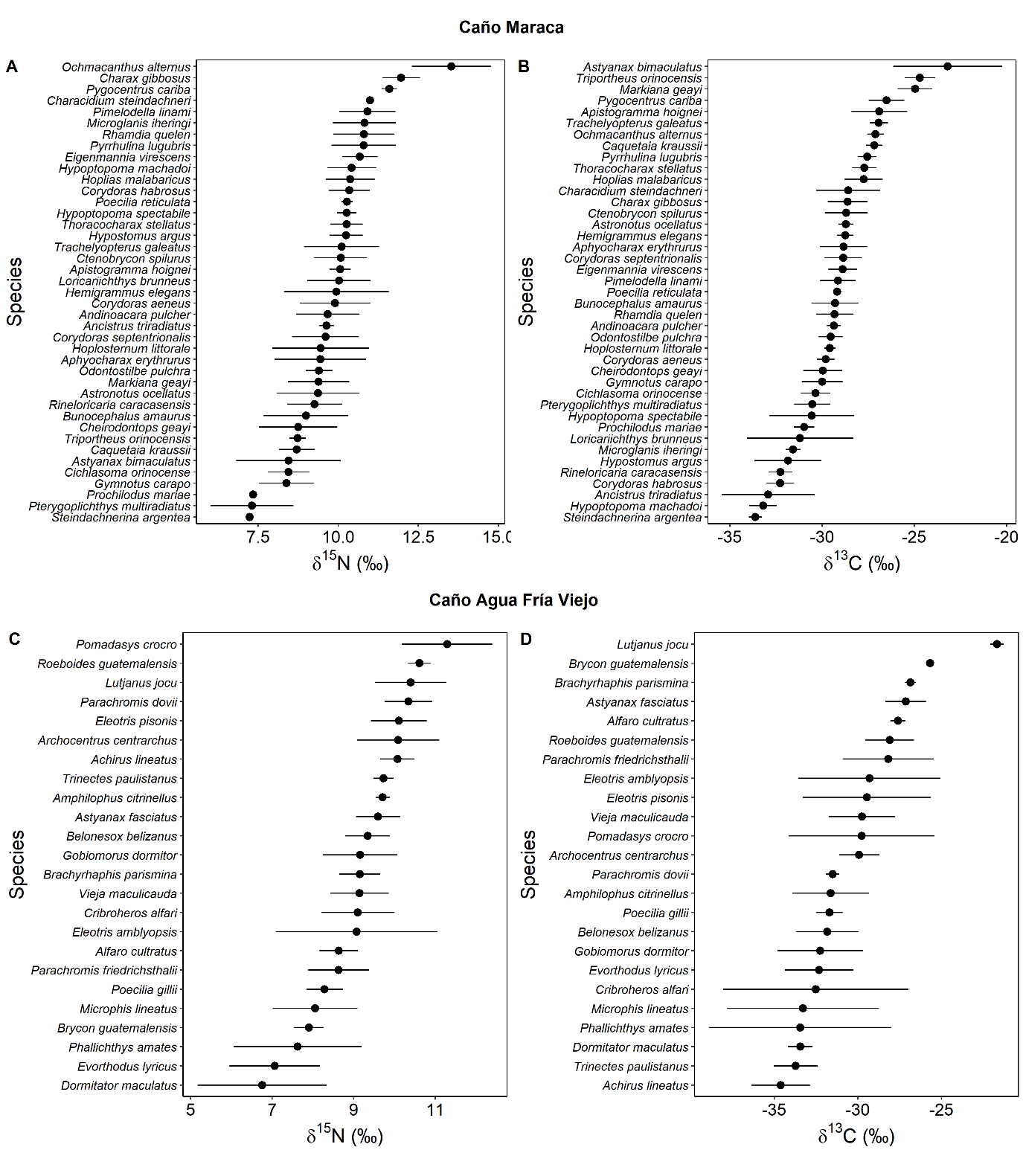

Supplement: Supplementary file 1 — Fig S1 [file ECE3-10-6562-s001.docx]
