# Supplementary material for: Can ancestry and morphology be used as surrogates for species niche relationships?
Source: Ecol Evol. 2020 Jun 3;10(13):6562–78. doi: 10.1002/ece3.6390 (PMC7381567; doi:10.1002/ece3.6390)

Figure S1. Example of the method used to account for the hierarchy nature of dietary data. The example consists of nine food items that are registered for five fish species. Two fish species are invertivores (Sp1 and Sp2), one is piscivorous (Sp3), and two are detritivores (Sp4 and Sp5). Some food items, such as invertebrates and fishes, are identified at lower taxonomic level, while others, such as detritus, are restricted to more broad categories. First (A), food items are organized according to their similarities into four hierarchical levels (L1, L2, L3, L4). Food item categories that have poor identification resolution and are from distinct clades are kept in levels of lower taxonomic resolution (bottom of the pyramid; A), which is represented by the dotted lines. For each hierarchical level, a diet matrix (consumers in rows and food item categories in columns) is created (B). These diet matrices were transformed into similarity matrices using Bray-Curtis dissimilarity (B) and then averaged, forming a unified similarity matrix that summarize the food overlap among species at the five resolution scales (C). This uniformed matrix can be used in hierarchical cluster analysis (D) or other multivariate analysis to visualize the diet overlap patterns among species.


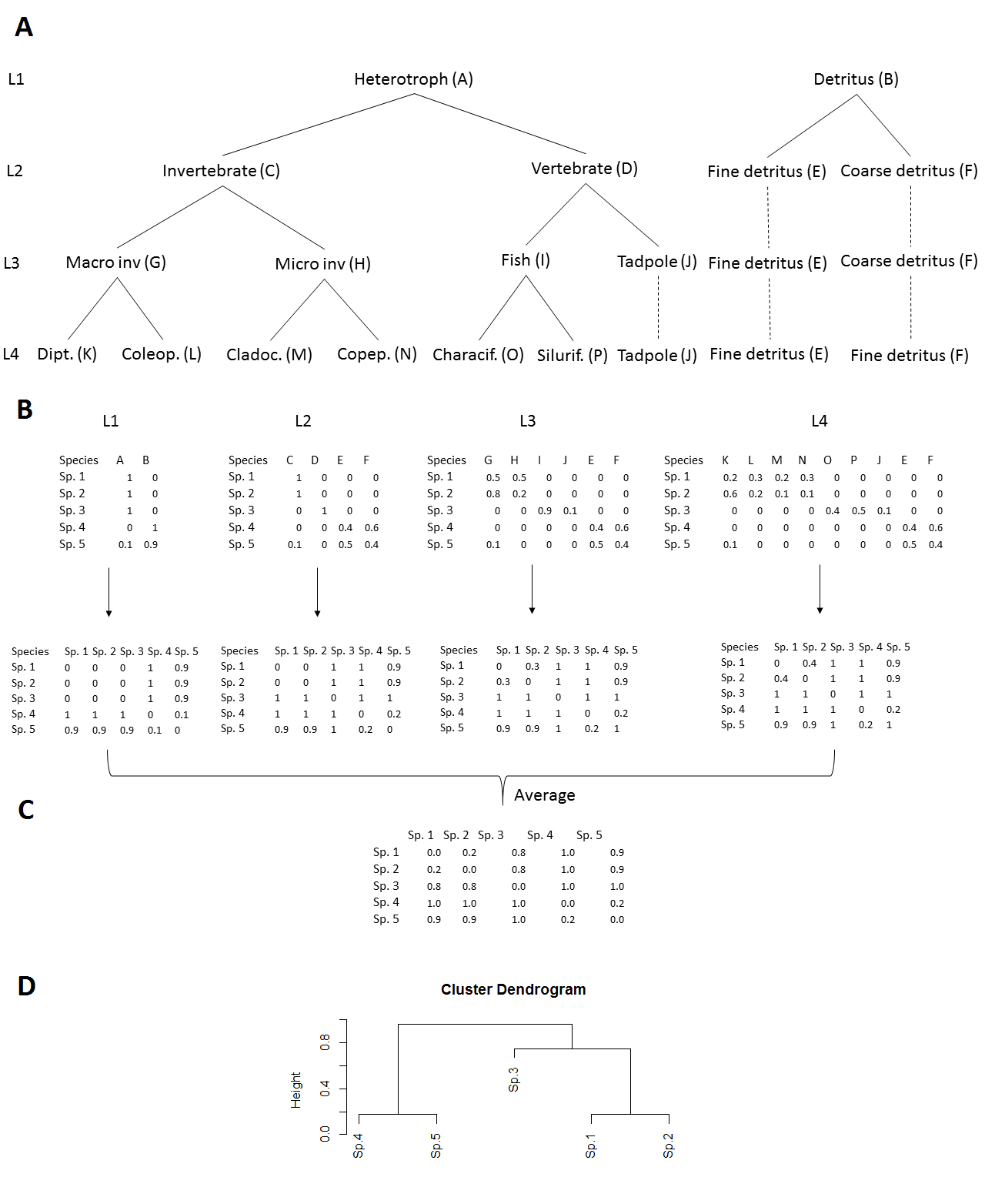

Supplement: Supplementary file 2 — Fig S2 [file ECE3-10-6562-s002.docx]
