# Supplementary material for: Can ancestry and morphology be used as surrogates for species niche relationships?
Source: Ecol Evol. 2020 Jun 3;10(13):6562–78. doi: 10.1002/ece3.6390 (PMC7381567; doi:10.1002/ece3.6390)

Figure S2. Tanglegrams constructed for pairwise comparisons of dendrograms based on phylogeny, morphological traits, diet, and stable isotopic ratios. Dendrograms were constructed using the UPGMA algorithm and using different species composition (species from Caño Maraca and species from Caño Agua Fría Viejo). We used an untangle function (algorithm step2side) to improve the visualization of the tanglegrams. Colors represent either different taxonomic orders or trophic groups.


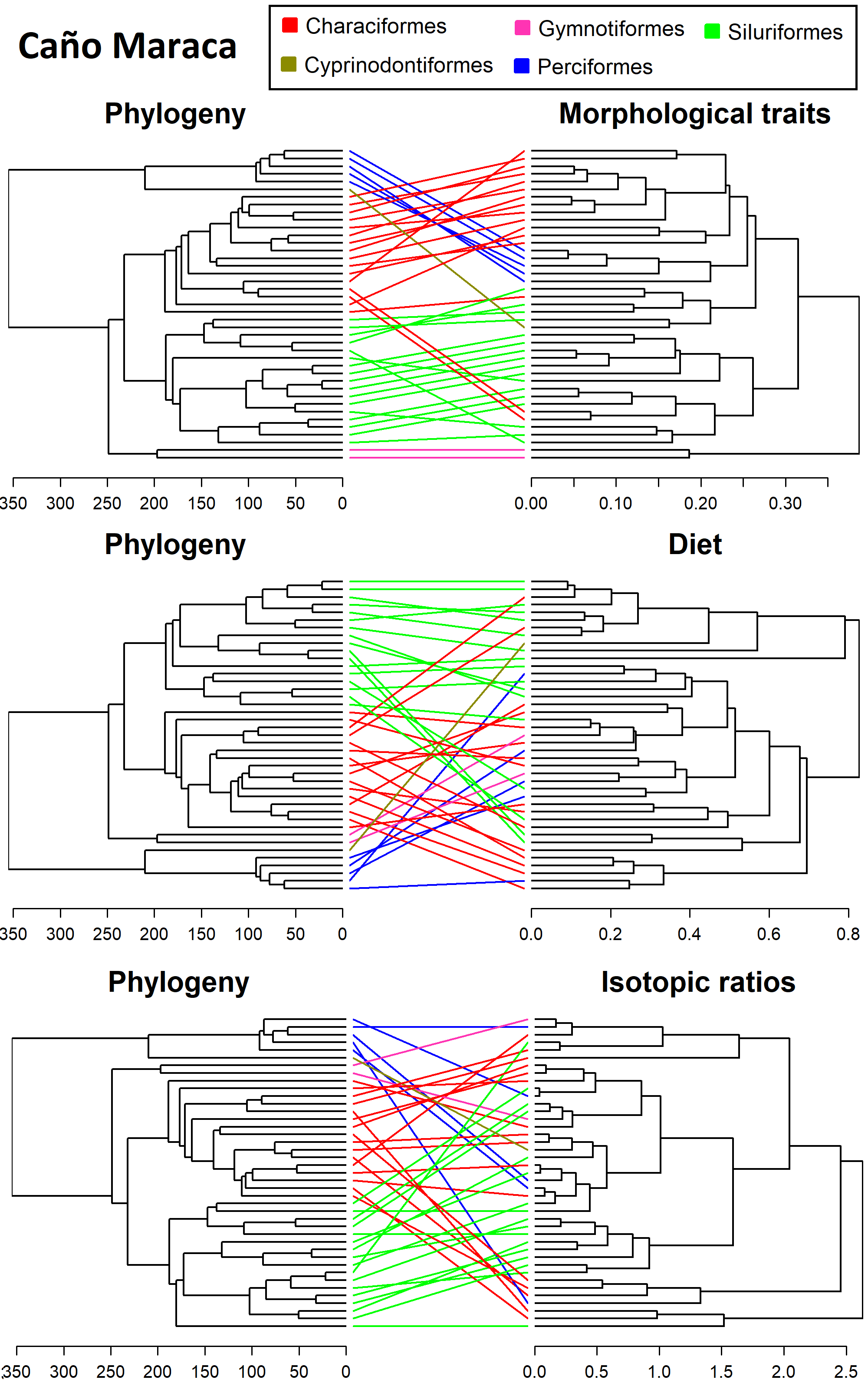


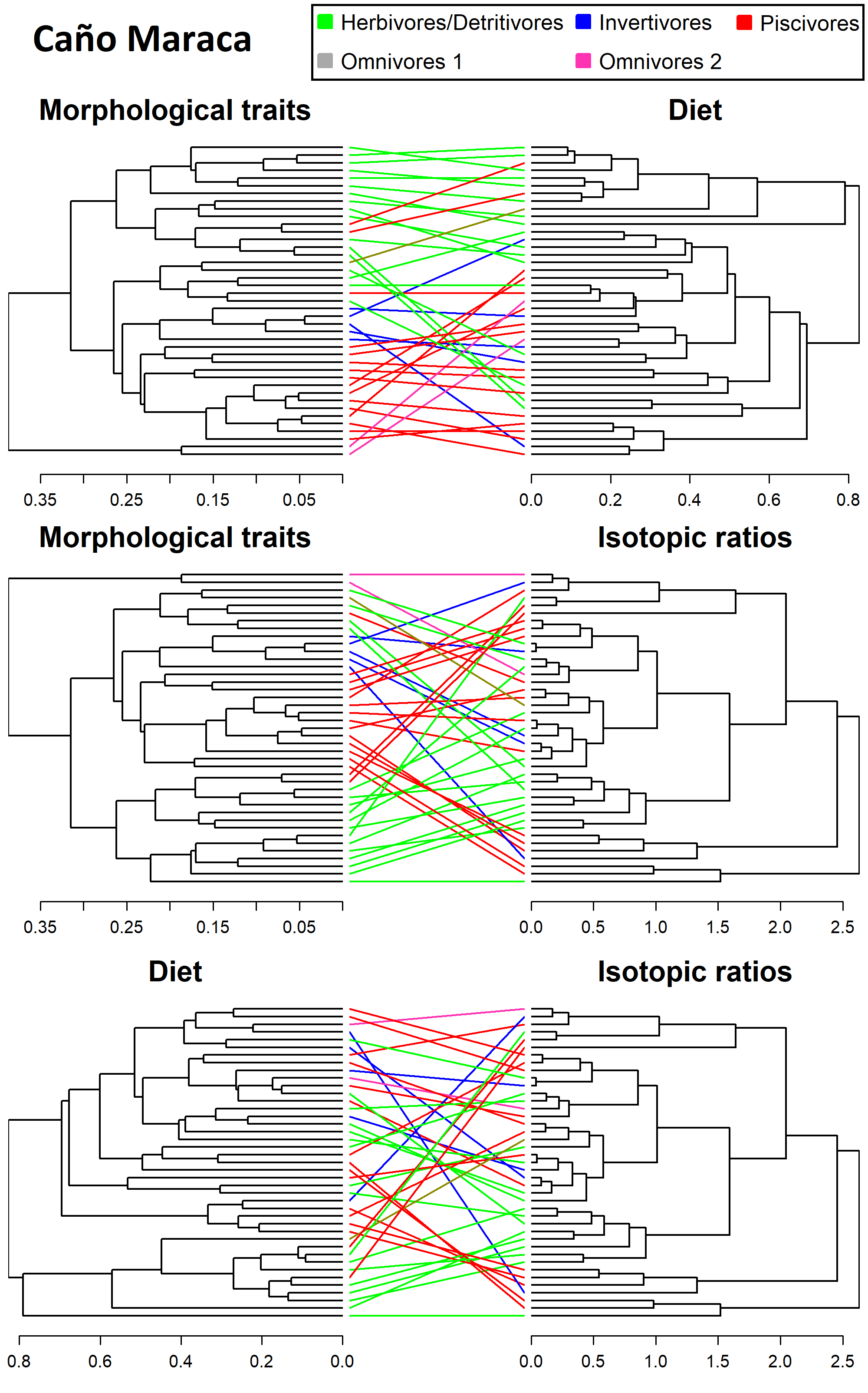


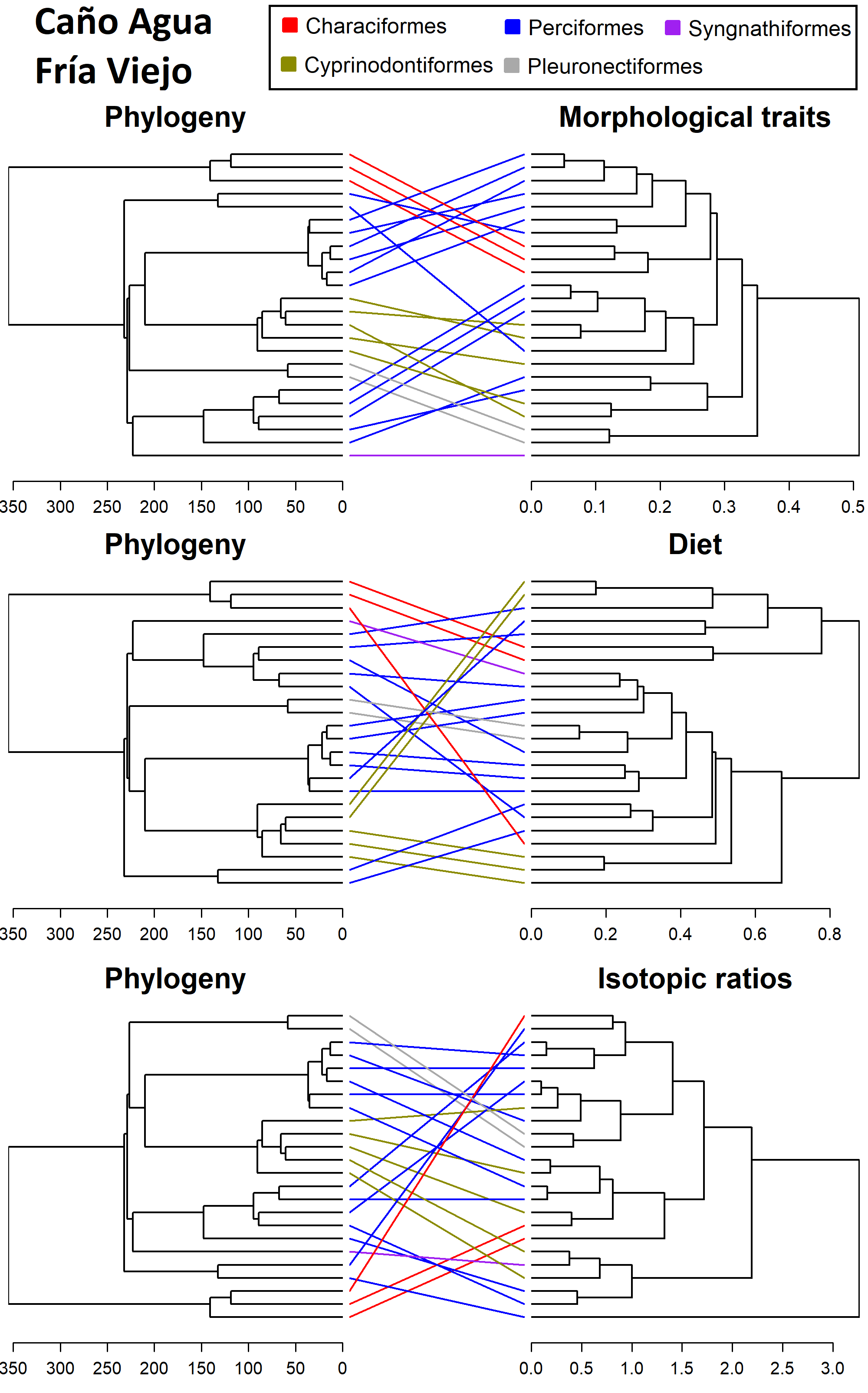


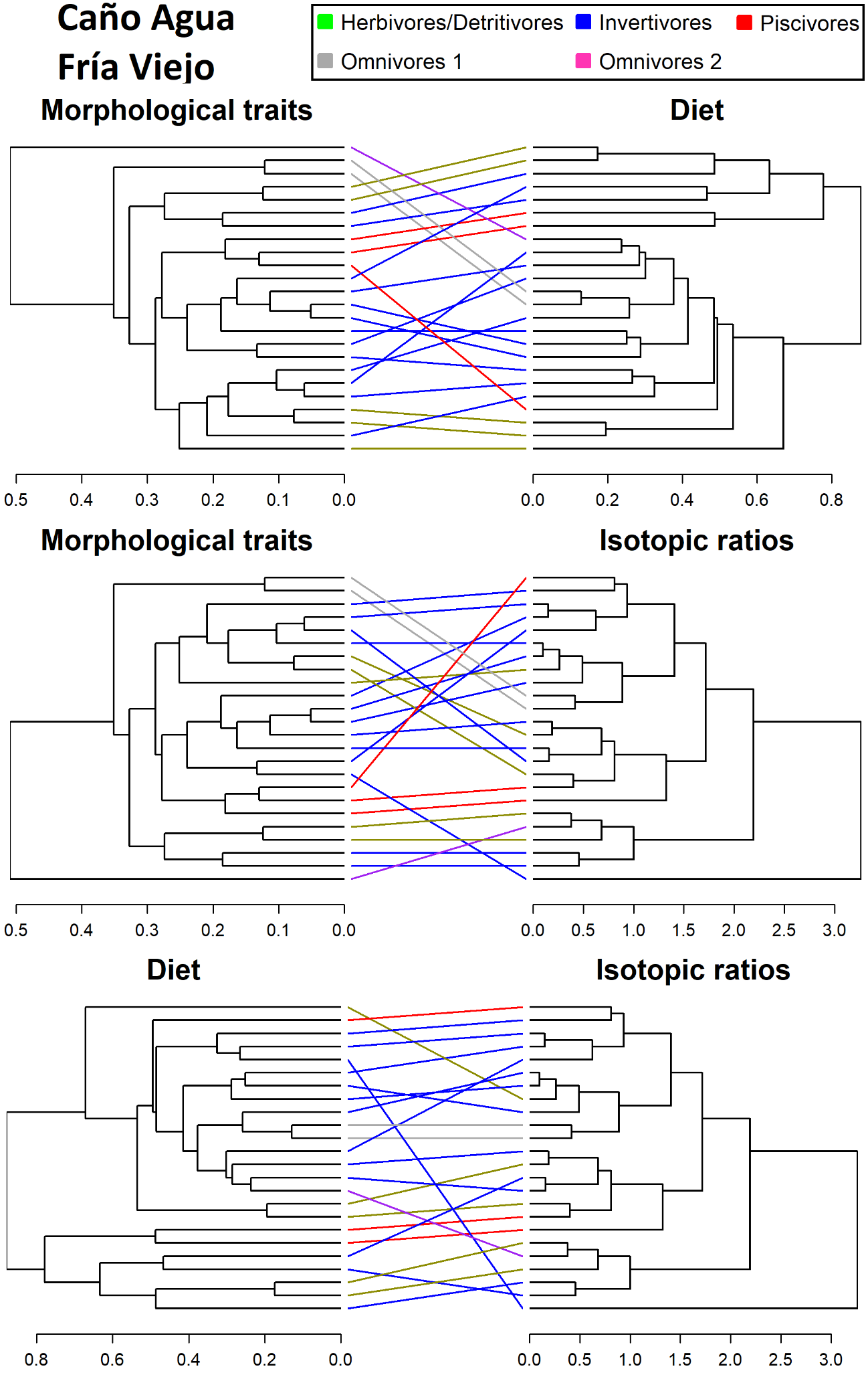

Supplement: Supplementary file 3 — Fig S3 [file ECE3-10-6562-s003.docx]
