# Supplementary material for: Can ancestry and morphology be used as surrogates for species niche relationships?
Source: Ecol Evol. 2020 Jun 3;10(13):6562–78. doi: 10.1002/ece3.6390 (PMC7381567; doi:10.1002/ece3.6390)

Figure S3. Phylogenetic signal calculated for morphological, diet, and isotopic dendrograms. Dendograms were constructured using the UPGMA algorithm and using different species composition (species from ALL sites combined; and from Caño Maraca and Caño Agua Fría Viejo separately). The method used to calculate the phylogenetic signals was similar to the one used by Cachera and Le Loc’h (2017), which is based on quantitative states generated for each tip of each dendrogram using Brownian simulations. These quantitative states are then tested for phylogenetic signal using the Abouheif's C mean index. Distribution of phylogenetic signal values for each dendrogram is based on 10,000 Brownian simulations. Abouheif’s *C*_mean_ varies from -1 (no phylogenetic signal) to 1 (complete phylogenetic signal).


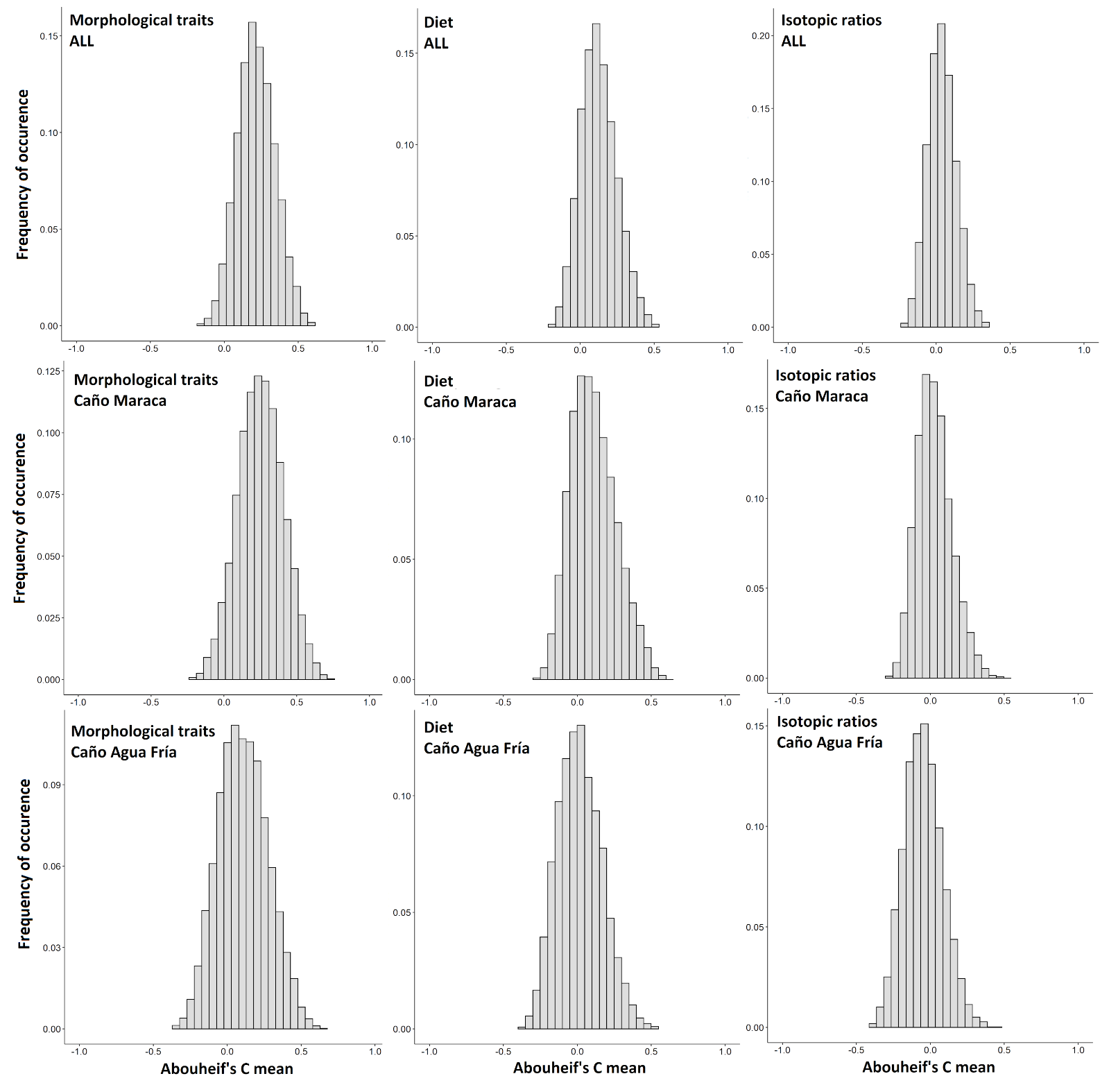

Supplement: Supplementary file 4 — Fig S4 [file ECE3-10-6562-s004.docx]
