# Supplementary material for: Can ancestry and morphology be used as surrogates for species niche relationships?
Source: Ecol Evol. 2020 Jun 3;10(13):6562–78. doi: 10.1002/ece3.6390 (PMC7381567; doi:10.1002/ece3.6390)
Supplement: Supplementary file 5 — Table S1 [file ECE3-10-6562-s005.docx]

Table S1. List of the fish species collected in Caño Maraca and Caño Agra Fría Viejo that were used for comparisons between phylogenic, morphological traits, dietary, and stable-isotope ratios datasets. Body size (mean and associated standard deviation) and number of individuals for diet, isotopic ratios and morphological traits are provided for each species.

| **Species** | **Site** | **N. of ind. analyzed for diet** | **N. of ind. analyzed for stable isotopes** | **N. of ind. analyzed for morphological traits** | **Size and SD of ind. analysed for diet** | **Size and SD of ind. analysed for stable isotopes** | **Size and SD of ind. analysed for morphological traits** |
| --- | --- | --- | --- | --- | --- | --- | --- |
| *Achirus lineatus* | Agua Fría Viejo | 17 | 4 | 9 | 42.42 (11.16) | 42.12 (1.82) | 65.13 (18.77) |
| *Alfaro cultratus* | Agua Fría Viejo | 262 | 3 | 3 | 34.08 (10.46) | 35.73 (1.63) | 51.83 (6.24) |
| *Amphilophus citrinellus* | Agua Fría Viejo | 45 | 3 | 3 | 71.62 (68.27) | 44 (25.64) | 109.73 (22.03) |
| *Ancistrus triradiatus* | Maraca | 68 | 4 | 3 | 44.78 (13.72) | 61.52 (6.47) | 69.63 (9.55) |
| *Andinoacara pulcher* | Maraca | 396 | 3 | 5 | 44.08 (17.22) | 23.17 (3.34) | 61.54 (9.08) |
| *Aphyocharax erythrurus* | Maraca | 192 | 3 | 3 | 30.27 (3.11) | 33.6 (0.7) | 32.53 (4.05) |
| *Apistogramma hoignei* | Maraca | 163 | 3 | 3 | 22.24 (3.84) | 26.5 (2.43) | 26.07 (1.75) |
| *Archocentrus centrarchus* | Agua Fría Viejo | 82 | 3 | 3 | 29.09 (16.37) | 20.83 (0.84) | 67 (9.6) |
| *Astronotus ocellatus* | Maraca | 53 | 2 | 3 | 130.41 (34.71) | 167.5 (4.95) | 152.67 (36.94) |
| *Astyanax bimaculatus* | Maraca | 203 | 3 | 6 | 45.17 (9.34) | 49.1 (0.95) | 45.68 (8.82) |
| *Astyanax fasciatus* | Agua Fría Viejo | 275 | 3 | 3 | 47.02 (18.63) | 46.4 (1.68) | 62.13 (12.11) |
| *Belonesox belizanus* | Agua Fría Viejo | 40 | 4 | 3 | 64.53 (32.34) | 70.58 (5.94) | 114.67 (9.83) |
| *Brachyhypopomus brevirostris* | Maraca | 30 | 3 | 3 | 44.31 (8.17) | 54.93 (3.56) | 192.17 (16.65) |
| *Brachyrhaphis parismina* | Agua Fría Viejo | 35 | 2 | 3 | 22.12 (6.26) | 28.9 (2.26) | 27.2 (7.71) |
| *Brycon guatemalensis* | Agua Fría Viejo | 60 | 3 | 3 | 156.52 (75.19) | 82.3 (10.65) | 165.6 (56.63) |
| *Bunocephalus amaurus* | Maraca | 109 | 3 | 5 | 38.4 (6.82) | 44.67 (2.72) | 53.52 (2.42) |
| *Caquetaia kraussii* | Maraca | 255 | 3 | 6 | 47.94 (25.56) | 34.1 (7.46) | 145.18 (38.39) |
| *Characidium steindachneri* | Maraca | 172 | 2 | 2 | 24.21 (2.06) | 26 (1.41) | 18.65 (2.19) |
| *Charax gibbosus* | Maraca | 149 | 3 | 3 | 56.87 (16.81) | 107.7 (16.37) | 69.77 (12.74) |
| *Cheirodontops geayi* | Maraca | 88 | 3 | 6 | 23.03 (2.34) | 27.33 (0.61) | 27.02 (2.78) |
| *Cichlasoma orinocense* | Maraca | 131 | 4 | 3 | 53.94 (20.77) | 59.85 (14.06) | 79.8 (18.8) |
| *Corydoras aeneus* | Maraca | 128 | 3 | 3 | 34.39 (3.45) | 37.63 (3.99) | 34.9 (3.7) |
| *Corydoras habrosus* | Maraca | 174 | 3 | 3 | 17.17 (2) | 17.4 (0.36) | 19.2 (0.8) |
| *Corydoras septentrionalis* | Maraca | 112 | 3 | 3 | 33.4 (4.73) | 39.5 (2.17) | 39.2 (1.71) |
| *Cribroheros alfari* | Agua Fría Viejo | 16 | 2 | 3 | 43.91 (35.34) | 94.85 (9.97) | 109.73 (19.86) |
| *Ctenobrycon spilurus* | Maraca | 233 | 3 | 3 | 37.43 (3.31) | 38.8 (2.29) | 38.3 (2.93) |
| *Dormitator maculatus* | Agua Fría Viejo | 107 | 3 | 3 | 45.77 (12.95) | 41.83 (0.99) | 62.1 (3.31) |
| *Eigenmannia virescens* | Maraca | 115 | 6 | 3 | 98.51 (21.01) | 154.6 (10.71) | 112.67 (6.51) |
| *Eleotris amblyopsis* | Agua Fría Viejo | 263 | 3 | 3 | 44.94 (14.4) | 71.4 (7.31) | 71.17 (7.24) |
| *Eleotris pisonis* | Agua Fría Viejo | 30 | 3 | 3 | 79.03 (32.92) | 101.43 (16.07) | 97.73 (14.32) |
| *Evorthodus lyricus* | Agua Fría Viejo | 23 | 7 | 3 | 32.31 (10.28) | 31.47 (12.17) | 42.9 (9.76) |
| *Gobiomorus dormitor* | Agua Fría Viejo | 77 | 6 | 3 | 59.11 (41.31) | 69.25 (40.57) | 176.73 (26.6) |
| *Gymnotus carapo* | Maraca | 195 | 5 | 3 | 166.32 (61.27) | 167.32 (26.64) | 162.5 (11.46) |
| *Hemigrammus elegans* | Maraca | 98 | 3 | 6 | 21.13 (3.03) | 23.83 (4.2) | 25.32 (4.71) |
| *Hoplias malabaricus* | Maraca | 191 | 3 | 3 | 77.63 (61.8) | 140.3 (12.92) | 166.67 (32.81) |
| *Hoplosternum littorale* | Maraca | 26 | 3 | 3 | 116.2 (24.51) | 92.13 (3.76) | 105.87 (11.57) |
| *Hypoptopoma spectabile* | Maraca | 71 | 3 | 4 | 21.2 (2.4) | 25.87 (1.53) | 45.12 (7.78) |
| *Hypostomus argus* | Maraca | 53 | 3 | 3 | 104.69 (43.41) | 65.13 (4.37) | 132 (39.13) |
| *Loricariichthys brunneus* | Maraca | 39 | 2 | 3 | 169.12 (43.7) | 186.5 (13.44) | 163.5 (42.39) |
| *Lutjanus jocu* | Agua Fría Viejo | 21 | 2 | 3 | 145.49 (37.26) | 102.95 (6.29) | 120.43 (9.58) |
| *Markiana geayi* | Maraca | 148 | 3 | 5 | 61.81 (7.79) | 68.73 (1.6) | 65.56 (2.78) |
| *Microglanis iheringi* | Maraca | 111 | 3 | 3 | 26.66 (3.43) | 35.07 (2.21) | 27.03 (2.06) |
| *Microphis lineatus* | Agua Fría Viejo | 72 | 3 | 3 | 100.59 (20.54) | 122.53 (2.2) | 133 (26.06) |
| *Ochmacanthus alternus* | Maraca | 193 | 3 | 3 | 29.55 (3.7) | 33.87 (3.27) | 33.93 (1.52) |
| *Odontostilbe pulchra* | Maraca | 228 | 3 | 3 | 24.68 (2.26) | 26.2 (1.47) | 27.47 (1.85) |
| *Parachromis dovii* | Agua Fría Viejo | 89 | 2 | 3 | 55.63 (62.93) | 99.1 (50.06) | 158.47 (19.63) |
| *Parachromis friedrichsthalii* | Agua Fría Viejo | 232 | 3 | 3 | 49.27 (30.7) | 71.97 (25.02) | 114.4 (26.61) |
| *Phallichthys amates* | Agua Fría Viejo | 181 | 3 | 3 | 33.96 (8.3) | 44.67 (1.85) | 49.53 (4.45) |
| *Pimelodella linami* | Maraca | 123 | 3 | 3 | 44.2 (6.86) | 55.63 (5.95) | 71.43 (6.1) |
| *Poecilia gillii* | Agua Fría Viejo | 143 | 3 | 3 | 48.56 (17.91) | 65.23 (2.85) | 70.87 (6.79) |
| *Poecilia reticulata* | Maraca | 145 | 3 | 3 | 18.71 (3.68) | 17.37 (1.25) | 20.4 (1.57) |
| *Pomadasys crocro* | Agua Fría Viejo | 54 | 3 | 3 | 125.15 (100.16) | 66.1 (14.82) | 106.8 (59.94) |
| *Prochilodus mariae* | Maraca | 49 | 3 | 3 | 147.39 (38.13) | 104 (3.52) | 109.7 (7.1) |
| *Pterygoplichthys multiradiatus* | Maraca | 34 | 3 | 3 | 167.53 (50.3) | 83.67 (0.45) | 117 (41.94) |
| *Pygocentrus cariba* | Maraca | 143 | 3 | 3 | 92.43 (20.18) | 102.87 (4.86) | 103.17 (8.56) |
| *Pyrrhulina lugubris* | Maraca | 154 | 3 | 5 | 32.17 (2.9) | 30.97 (0.25) | 35.34 (2.32) |
| *Rhamdia quelen* | Maraca | 86 | 3 | 3 | 100.32 (17.35) | 93.6 (6.32) | 100.47 (13.15) |
| *Rineloricaria caracasensis* | Maraca | 41 | 3 | 3 | 82.4 (18.59) | 77.83 (2.23) | 104.1 (0.36) |
| *Roeboides guatemalensis* | Agua Fría Viejo | 69 | 3 | 3 | 63.6 (13.49) | 72.13 (4.3) | 69.4 (3.41) |
| *Steindachnerina argentea* | Maraca | 64 | 3 | 3 | 51.09 (9.45) | 54.7 (4.37) | 61.43 (7.31) |
| *Thoracocharax stellatus* | Maraca | 177 | 3 | 3 | 29.72 (3.47) | 30.27 (1.8) | 32.6 (1.85) |
| *Trachelyopterus galeatus* | Maraca | 147 | 3 | 3 | 72.72 (11.84) | 92.6 (8.11) | 69.23 (3.16) |
| *Trinectes paulistanus* | Agua Fría Viejo | 18 | 3 | 3 | 32.04 (12.79) | 46.6 (9.81) | 54.3 (3.21) |
| *Triportheus orinocensis* | Maraca | 157 | 3 | 3 | 62.27 (13.85) | 78.6 (9.8) | 88.97 (37.27) |
| *Vieja maculicauda* | Agua Fría Viejo | 65 | 3 | 3 | 118.06 (62.76) | 116.83 (38.98) | 137 (12.53) |
