# Supplementary material for: Can ancestry and morphology be used as surrogates for species niche relationships?
Source: Ecol Evol. 2020 Jun 3;10(13):6562–78. doi: 10.1002/ece3.6390 (PMC7381567; doi:10.1002/ece3.6390)
Supplement: Supplementary file 6 — Table S2 [file ECE3-10-6562-s006.docx]

Table S2: Food item categories organized in seven hierarchical levels, from course (Level 1) to fine taxonomic resolution (Level 7). Number of categories per level is provided in parenthesis.

| **Level 1 (# Cat: 3)** | **Level 2 (# Cat: 7)** | **Level 3 (# Cat: 16)** | **Level 4 (# Cat: 30)** | **Level 5 (# Cat: 66)** | **Level 6 (# Cat: 90)** | **Level 7 (# Cat: 126)** |
| --- | --- | --- | --- | --- | --- | --- |
| Autotrophs | Plants | Veg. plant structures | Veg. aquatic structures | Veg. aquatic structures | Veg. aquatic structures | Veg. aquatic structures |
| Autotrophs | Plants | Veg. plant structures | Veg. terrestrial structures | Veg. terrestrial structures | Veg. terrestrial structures | Veg. terrestrial structures |
| Autotrophs | Plants | Soft flowers and fruits | Soft flowers and fruits | Soft flowers and fruits | Soft flowers and fruits | Soft flowers and fruits |
| Autotrophs | Plants | Hard fruits and seeds | Hard fruits and seeds | Hard fruits and seeds | Hard fruits and seeds | Hard fruits and seeds |
| Autotrophs | Plants | Plant spores | Plant Spores | Plant Spores | Plant Spores | Plant Spores |
| Autotrophs | Algae | Multicelular algae | Chara | Chara | Chara | Chara |
| Autotrophs | Algae | Multicelular algae | Filamentous algae | Filamentous algae | Filamentous algae | Filamentous algae |
| Autotrophs | Algae | Diatoms | Diatoms | Diatoms | Diatoms | Diatoms |
| Autotrophs | Algae | Desmids | Desmids | Desmids | Desmids | Desmids |
| Detritus | Fine detritus | Fine detritus | Fine detritus | Fine detritus | Fine detritus | Fine detritus |
| Detritus | Vegetative detritus | Vegetative detritus | Vegetative detritus | Vegetative detritus | Vegetative detritus | Vegetative detritus |
| Detritus | Coarse detritus | Coarse detritus | Coarse detritus | Coarse detritus | Coarse detritus | Coarse detritus |
| Heterotrophs | Invertebrates | Invertebrate eggs | Invertebrate eggs | Invertebrate eggs | Invertebrate eggs | Invertebrate eggs |
| Heterotrophs | Invertebrates | Microinvertebrates | Protozoa | Protozoa | Protozoa | Protozoa |
| Heterotrophs | Invertebrates | Microinvertebrates | Rotifers | Rotifers | Rotifers | Rotifers |
| Heterotrophs | Invertebrates | Microinvertebrates | Rotifers | Diflugiids | Diflugiids | Diflugiids |
| Heterotrophs | Invertebrates | Microinvertebrates | Microcrustacea | Copepoda | Copepoda | Copepoda |
| Heterotrophs | Invertebrates | Microinvertebrates | Microcrustacea | Cladocera | Cladocera | Cladocera |
| Heterotrophs | Invertebrates | Microinvertebrates | Microcrustacea | Ostracoda | Ostracoda | Ostracoda |
| Heterotrophs | Invertebrates | Macroinvertebrates | Nematoda | Nematoda | Nematoda | Nematoda |
| Heterotrophs | Invertebrates | Macroinvertebrates | Nematophora | Nematophora | Nematophora | Nematophora |
| Heterotrophs | Invertebrates | Macroinvertebrates | Annelida | Oligochaeta | Oligochaeta | Oligochaeta |
| Heterotrophs | Invertebrates | Macroinvertebrates | Annelida | Hirudinea | Hirudinea | Hirudinea |
| Heterotrophs | Invertebrates | Macroinvertebrates | Mollusca | Gastropoda | Gastropoda | Gastropoda |
| Heterotrophs | Invertebrates | Macroinvertebrates | Mollusca | Bivalve | Bivalve | Bivalve |
| Heterotrophs | Invertebrates | Macroinvertebrates | Macrocrustaceans | Anostraca (fairy shrimp) | Anostraca (fairy shrimp) | Anostraca (fairy shrimp) |
| Heterotrophs | Invertebrates | Macroinvertebrates | Macrocrustaceans | Caridea (shrimps) | Caridea (shrimps) | Caridea (shrimps) |
| Heterotrophs | Invertebrates | Macroinvertebrates | Macrocrustaceans | Brachyura (crabs) | Brachyura (crabs) | Brachyura (crabs) |
| Heterotrophs | Invertebrates | Macroinvertebrates | Macrocrustaceans | Isopoda | Isopoda | Isopoda |
| Heterotrophs | Invertebrates | Macroinvertebrates | Macrocrustaceans | Amphipoda | Amphipoda | Amphipoda |
| Heterotrophs | Invertebrates | Macroinvertebrates | Collembola | Collembola | Collembola | Collembola |
| Heterotrophs | Invertebrates | Macroinvertebrates | Chelicerata | Acarina | Acarina | Acarina |
| Heterotrophs | Invertebrates | Macroinvertebrates | Chelicerata | Araneae | Araneae | Araneae |
| Heterotrophs | Invertebrates | Macroinvertebrates | Insects | Diptera (larvae) | Chironomidae (larvae) | Chironomidae (larvae) |
| Heterotrophs | Invertebrates | Macroinvertebrates | Insects | Diptera (larvae) | Other Diptera (larvae) | Other Diptera (larvae) |
| Heterotrophs | Invertebrates | Macroinvertebrates | Insects | Diptera (larvae) | Mosquito (larvae) | Mosquito (larvae) |
| Heterotrophs | Invertebrates | Macroinvertebrates | Insects | Diptera (adult) | Diptera (adult) | Diptera (adult) |
| Heterotrophs | Invertebrates | Macroinvertebrates | Insects | Odonata (nymph) | Odonata (nymph) | Odonata (nymph) |
| Heterotrophs | Invertebrates | Macroinvertebrates | Insects | Ephemeroptera | Ephemeroptera | Ephemeroptera |
| Heterotrophs | Invertebrates | Macroinvertebrates | Insects | Coleoptera (aquatic larvae) | Coleoptera (aquatic larvae) | Coleoptera (aquatic larvae) |
| Heterotrophs | Invertebrates | Macroinvertebrates | Insects | Coleoptera (aquatic adult) | Coleoptera (aquatic adult) | Coleoptera (aquatic adult) |
| Heterotrophs | Invertebrates | Macroinvertebrates | Insects | Coleoptera (terrestrial adult) | Coleoptera (terrestrial adult) | Coleoptera (terrestrial adult) |
| Heterotrophs | Invertebrates | Macroinvertebrates | Insects | Plecoptera | Plecoptera | Plecoptera |
| Heterotrophs | Invertebrates | Macroinvertebrates | Insects | Trichoptera | Trichoptera | Trichoptera |
| Heterotrophs | Invertebrates | Macroinvertebrates | Insects | Neuroptera | Neuroptera | Neuroptera |
| Heterotrophs | Invertebrates | Macroinvertebrates | Insects | Orthoptera | Orthoptera | Orthoptera |
| Heterotrophs | Invertebrates | Macroinvertebrates | Insects | Hemiptera (aquatic) | Hemiptera (aquatic) | Hemiptera (aquatic) |
| Heterotrophs | Invertebrates | Macroinvertebrates | Insects | Hemiptera (aquatic) | Corixidae | Corixidae |
| Heterotrophs | Invertebrates | Macroinvertebrates | Insects | Hemiptera (aquatic) | Gerridae | Gerridae |
| Heterotrophs | Invertebrates | Macroinvertebrates | Insects | Hemiptera (terrestrial) | Hemiptera (terrestrial) | Hemiptera (terrestrial) |
| Heterotrophs | Invertebrates | Macroinvertebrates | Insects | Lepidoptera (larvae) | Lepidoptera (larvae) | Lepidoptera (larvae) |
| Heterotrophs | Invertebrates | Macroinvertebrates | Insects | Lepidoptera (adult) | Lepidoptera (adult) | Lepidoptera (adult) |
| Heterotrophs | Invertebrates | Macroinvertebrates | Insects | Hymenoptera | Hymenoptera | Hymenoptera |
| Heterotrophs | Invertebrates | Macroinvertebrates | Insects | Isoptera | Isoptera | Isoptera |
| Heterotrophs | Vertebrates | Non-fish vertebrates | Anura | Anura (tadpoles) | Anura (tadpoles) | Anura (tadpoles) |
| Heterotrophs | Vertebrates | Non-fish vertebrates | Anura | Anura (frogs) | Anura (frogs) | Anura (frogs) |
| Heterotrophs | Vertebrates | Fish eggs | Fish eggs | Fish eggs | Fish eggs | Fish eggs |
| Heterotrophs | Vertebrates | Fish | Fish mucus | Fish mucus | Fish mucus | Fish mucus |
| Heterotrophs | Vertebrates | Fish | Fish scales | Fish scales | Fish scales | Fish scales |
| Heterotrophs | Vertebrates | Fish | Fish fins | Fish fins | Fish fins | Fish fins |
| Heterotrophs | Vertebrates | Fish | Fish (whole) | Atheriniformes | Atherinopsidae | Atherinella |
| Heterotrophs | Vertebrates | Fish | Fish (whole) | Characiformes | Anostomidae | Schizodon |
| Heterotrophs | Vertebrates | Fish | Fish (whole) | Characiformes | Bryconidae | Brycon |
| Heterotrophs | Vertebrates | Fish | Fish (whole) | Characiformes | Characidae | Aphyocharax |
| Heterotrophs | Vertebrates | Fish | Fish (whole) | Characiformes | Characidae | Astyanax |
| Heterotrophs | Vertebrates | Fish | Fish (whole) | Characiformes | Characidae | Bryconamericus |
| Heterotrophs | Vertebrates | Fish | Fish (whole) | Characiformes | Characidae | Cheirodontops |
| Heterotrophs | Vertebrates | Fish | Fish (whole) | Characiformes | Characidae | Corynopoma |
| Heterotrophs | Vertebrates | Fish | Fish (whole) | Characiformes | Characidae | Creagrutus |
| Heterotrophs | Vertebrates | Fish | Fish (whole) | Characiformes | Characidae | Ctenobrycon |
| Heterotrophs | Vertebrates | Fish | Fish (whole) | Characiformes | Characidae | Gephyrocharax |
| Heterotrophs | Vertebrates | Fish | Fish (whole) | Characiformes | Characidae | Hemigrammus |
| Heterotrophs | Vertebrates | Fish | Fish (whole) | Characiformes | Characidae | Markiana |
| Heterotrophs | Vertebrates | Fish | Fish (whole) | Characiformes | Characidae | Odontostilbe |
| Heterotrophs | Vertebrates | Fish | Fish (whole) | Characiformes | Characidae | Poptella |
| Heterotrophs | Vertebrates | Fish | Fish (whole) | Characiformes | Characidae | Roeboides |
| Heterotrophs | Vertebrates | Fish | Fish (whole) | Characiformes | Characidae | Tetragonopterus |
| Heterotrophs | Vertebrates | Fish | Fish (whole) | Characiformes | Crenuchidae | Characidium |
| Heterotrophs | Vertebrates | Fish | Fish (whole) | Characiformes | Curimatidae | Curimata |
| Heterotrophs | Vertebrates | Fish | Fish (whole) | Characiformes | Erythrinidae | Hoplias |
| Heterotrophs | Vertebrates | Fish | Fish (whole) | Characiformes | Gasteropelecidae | Thoracocharax |
| Heterotrophs | Vertebrates | Fish | Fish (whole) | Characiformes | Lebiasinidae | Pyrrhulina |
| Heterotrophs | Vertebrates | Fish | Fish (whole) | Characiformes | Prochilodontidae | Prochilodus |
| Heterotrophs | Vertebrates | Fish | Fish (whole) | Characiformes | Serrasalmidae | Pygocentrus |
| Heterotrophs | Vertebrates | Fish | Fish (whole) | Characiformes | Triportheidae | Triportheus |
| Heterotrophs | Vertebrates | Fish | Fish (whole) | Clupeiformes | Engraulidae | Anchoviella |
| Heterotrophs | Vertebrates | Fish | Fish (whole) | Cyprinodontiformes | Cynolebiidae | Rachovia |
| Heterotrophs | Vertebrates | Fish | Fish (whole) | Cyprinodontiformes | Cynolebiidae | Rivulus |
| Heterotrophs | Vertebrates | Fish | Fish (whole) | Cyprinodontiformes | Poeciliidae | Alfaro |
| Heterotrophs | Vertebrates | Fish | Fish (whole) | Cyprinodontiformes | Poeciliidae | Belonesox |
| Heterotrophs | Vertebrates | Fish | Fish (whole) | Cyprinodontiformes | Poeciliidae | Phallichthys |
| Heterotrophs | Vertebrates | Fish | Fish (whole) | Cyprinodontiformes | Poeciliidae | Poecilia |
| Heterotrophs | Vertebrates | Fish | Fish (whole) | Gymnotiformes | Gymnotidae | Gymnotus |
| Heterotrophs | Vertebrates | Fish | Fish (whole) | Perciformes | Cichlidae | Aequidens |
| Heterotrophs | Vertebrates | Fish | Fish (whole) | Perciformes | Cichlidae | Amatitlania |
| Heterotrophs | Vertebrates | Fish | Fish (whole) | Perciformes | Cichlidae | Amphilophus |
| Heterotrophs | Vertebrates | Fish | Fish (whole) | Perciformes | Cichlidae | Apistogramma |
| Heterotrophs | Vertebrates | Fish | Fish (whole) | Perciformes | Cichlidae | Archocentrus |
| Heterotrophs | Vertebrates | Fish | Fish (whole) | Perciformes | Cichlidae | Astronotus |
| Heterotrophs | Vertebrates | Fish | Fish (whole) | Perciformes | Cichlidae | Caquetaia |
| Heterotrophs | Vertebrates | Fish | Fish (whole) | Perciformes | Cichlidae | Crenicichla |
| Heterotrophs | Vertebrates | Fish | Fish (whole) | Perciformes | Cichlidae | Parachromis |
| Heterotrophs | Vertebrates | Fish | Fish (whole) | Perciformes | Cichlidae | Vieja |
| Heterotrophs | Vertebrates | Fish | Fish (whole) | Perciformes | Eleotridae | Dormitator |
| Heterotrophs | Vertebrates | Fish | Fish (whole) | Perciformes | Eleotridae | Eleotris |
| Heterotrophs | Vertebrates | Fish | Fish (whole) | Perciformes | Eleotridae | Gobiomorus |
| Heterotrophs | Vertebrates | Fish | Fish (whole) | Perciformes | Gerreidae | Diapterus |
| Heterotrophs | Vertebrates | Fish | Fish (whole) | Perciformes | Gobiidae | Ctenogobius |
| Heterotrophs | Vertebrates | Fish | Fish (whole) | Perciformes | Haemulidae | Pomadasys |
| Heterotrophs | Vertebrates | Fish | Fish (whole) | Siluriformes | Aspredinidae | Bunocephalus |
| Heterotrophs | Vertebrates | Fish | Fish (whole) | Siluriformes | Auchenipteridae | Ageneiosus |
| Heterotrophs | Vertebrates | Fish | Fish (whole) | Siluriformes | Auchenipteridae | Entomocorus |
| Heterotrophs | Vertebrates | Fish | Fish (whole) | Siluriformes | Auchenipteridae | Parauchenipterus |
| Heterotrophs | Vertebrates | Fish | Fish (whole) | Siluriformes | Callichthyidae | Corydoras |
| Heterotrophs | Vertebrates | Fish | Fish (whole) | Siluriformes | Callichthyidae | Hoplosternum |
| Heterotrophs | Vertebrates | Fish | Fish (whole) | Siluriformes | Heptapteridae | Pimelodella |
| Heterotrophs | Vertebrates | Fish | Fish (whole) | Siluriformes | Heptapteridae | Rhamdia |
| Heterotrophs | Vertebrates | Fish | Fish (whole) | Siluriformes | Loricariidae | Ancistrus |
| Heterotrophs | Vertebrates | Fish | Fish (whole) | Siluriformes | Loricariidae | Hypostomus |
| Heterotrophs | Vertebrates | Fish | Fish (whole) | Siluriformes | Loricariidae | Loricarichthys |
| Heterotrophs | Vertebrates | Fish | Fish (whole) | Siluriformes | Loricariidae | Otocinclus |
| Heterotrophs | Vertebrates | Fish | Fish (whole) | Siluriformes | Loricariidae | Rineloricaria |
| Heterotrophs | Vertebrates | Fish | Fish (whole) | Siluriformes | Trichomycteridae | Ochmacanthus |
| Heterotrophs | Vertebrates | Fish | Fish (whole) | Synbranchiformes | Synbranchidae | Synbranchus |
| Heterotrophs | Vertebrates | Fish | Fish (whole) | Syngnathiformes | Syngnathidae | Oostethus |
