# Supplementary material for: Can ancestry and morphology be used as surrogates for species niche relationships?
Source: Ecol Evol. 2020 Jun 3;10(13):6562–78. doi: 10.1002/ece3.6390 (PMC7381567; doi:10.1002/ece3.6390)
Supplement: Supplementary file 7 — Table S3 [file ECE3-10-6562-s007.docx]

Table S3. Species that were not present in the super tree published by Rabosky et al. (2018). We included these unsampled species in the tree based on the position of related species or genus (right-side column).

| **Species** | **Position in the phylogenetic tree** |
| --- | --- |
| *Ancistrus triradiatus* | *Ancistrus multispinis* |
| *Aphyocharax erythrurus* | *Aphyocharax alburnus* |
| *Bunocephalus amaurus* | *Bunocephalus coracoideus* |
| *Characidium steindachneri* | *Characidium pterostictum* |
| *Charax gibbosus* | *Charax leticiae* |
| *Cheirodontops geayi* | *Cheirodon ibicuhiensis* |
| *Ctenobrycon spilurus* | *Ctenobrycon hauxwellianus* |
| *Hemigrammus elegans* | *Hemigrammus rodwayi* |
| *Hypostomus argus* | *Hypostomus plecostomoides* |
| *Lebiasina erythrinoides* | *Copella nattereri* |
| *Loricariichthys brunneus* | *Loricariichthys anus* |
| *Markiana geayi* | *Markiana nigripinnis* |
| *Pimelodella linami* | *Pimelodella lateristriga* |
| *Pyrrhulina lugubris* | *Pyrrhulina australis* |
| *Rineloricaria caracasensis* | *Rineloricaria parva* |
| *Steindachnerina argentea* | *Steindachnerina elegans* |
| *Brycon guatemalensis* | *Brycon hilarii* |
| *Microphis lineatus* | *Microphis brachyurus* |
| *Vieja maculicauda* | *Paraneetroplus maculicauda* |
| *Cribroheros alfari* | *Amphilophus alfari* |
| *Amatitlania septemfasciata* | *Cryptoheros septemfasciatus* |
